# Putative Role of Nuclear Factor-Kappa B But Not Hypoxia-Inducible Factor-1α in Hypoxia-Dependent Regulation of Oxidative Stress in Hematopoietic Stem and Progenitor Cells

**DOI:** 10.1089/ars.2018.7551

**Published:** 2019-06-20

**Authors:** Camilla Halvarsson, Emma Rörby, Pernilla Eliasson, Stefan Lang, Shamit Soneji, Jan-Ingvar Jönsson

**Affiliations:** ^1^ Experimental Hematology Unit, Department of Clinical and Experimental Medicine, Linköping University, Linköping, Sweden.; ^2^ Lund Stem Cell Center, Lund University, Lund, Sweden.

**Keywords:** hematopoiesis, hypoxia, oxidative stress, glutathione, mitochondria, NF-κB

## Abstract

***Aims:*** Adaptation to low oxygen of hematopoietic stem cells (HSCs) in the bone marrow has been demonstrated to depend on the activation of hypoxia-inducible factor (HIF)-1α as well as the limited production of reactive oxygen species (ROS). In this study, we aimed at determining whether HIF-1α is involved in protecting HSCs from ROS.

***Results:*** Oxidative stress was induced by DL-buthionine-(S,R)-sulfoximine (BSO)-treatment, which increases the mitochondrial ROS level. Hypoxia rescued Lineage-Sca-1^+^c-kit^+^ (LSK) cells from BSO-induced apoptosis, whereas cells succumbed to apoptosis in normoxia. Apoptosis in normoxia was inhibited with the antioxidant N-acetyl-L-cysteine or by overexpression of anti-apoptotic BCL-2. Moreover, stabilized expression of oxygen-insensitive HIFs could not protect LSK cells from oxidative stress-induced apoptosis at normoxia, neither could short hairpin RNA to *Hif-1α* inhibit the protective effects by hypoxia in LSK cells. Likewise, BSO treatment of LSK cells from *Hif-1α* knockout mice did not suppress the effects seen in hypoxia. Microarray analysis identified the nuclear factor-kappa B (NF-κB) pathway as a pathway induced by hypoxia. By using NF-κB lentiviral construct and DNA-binding assay, we found increased NF-κB activity in cells cultured in hypoxia compared with normoxia. Using an inhibitor against NF-κB activation, we could confirm the involvement of NF-κB signaling as BSO-mediated cell death was significantly increased in hypoxia after adding the inhibitor.

***Innovation:*** HIF-1α is not involved in protecting HSCs and progenitors to elevated levels of ROS on glutathione depletion during hypoxic conditions.

***Conclusion:*** The study proposes a putative role of NF-κB signaling as a hypoxia-induced regulator in early hematopoietic cells.

## Introduction

Hematopoietic stem cells (HSCs) are located in the bone marrow (BM). They are characterized by their ability to self-renew and differentiate into all blood cells. The BM is widely accepted as a relatively hypoxic tissue where HSCs reside mainly within niches of limited oxygen availability (36, 45, 59). Recent studies have demonstrated that the hypoxic environment of the BM is dependent on the high rate of oxygen molecule (O_2_) consumption and its dense vascularity (58). Thus, almost all HSCs, even those close to blood vessels, seem to have a hypoxic status (30, 38). Since most long-term (LT)-HSCs are dormant and low in metabolic activity, a low-oxygen environment is believed to sustain HSCs with slow cell cycling and a metabolic shift toward glycolysis (8, 54).

**Innovation**

Contrary to current suggestions that hypoxia protects hematopoietic stem cells (HSCs) and progenitors against oxidative stress by activation of hypoxia-inducible factor (HIF)-1α, this study shows that HIF-1α is dispensable for protection to elevated levels of reactive oxygen species (ROS). Thus, other molecular mechanisms must be essential for this hypoxia-mediated protection.

A balanced regulation of reactive oxygen species (ROS) is essential to maintain proper hematopoietic homeostasis and blood formation (64, 66), and intracellular ROS levels are kept low in LT-HSCs (23). In excess, different free radicals lead to oxidative stress characterized by damage to DNA, membrane lipids, and proteins. Excessive levels of ROS such as superoxide anion radicals (^•^O_2_^−^), hydroxyl radicals (^•^OH), and hydrogen peroxide (H_2_O_2_) have been reported to limit the lifespan of stem cells (19, 20, 23). To attenuate the generation of ROS, important self-defense mechanisms have evolved. In other cell systems, the redox balance has been shown to be maintained by the regulatory potential of molecular thiol-driven master switches such as nuclear factor erythroid 2-related factor 2 (NRF2), nuclear factor-kappa B (NF-κB), the methionine sulfoxide reductase system, and antioxidant enzymes such as catalase (CAT), glutathione (GSH), peroxidase, and superoxide dismutase (SOD) (2, 66).

In conditions of limited O_2_ supply, specific cellular sensors allow cells to adapt to the deprivation of oxygen. One of the best characterized sensors is hypoxia-inducible factors (HIFs), transcription factors that are central for the cellular adaptation to oxygen limitation as it upregulates genes that are involved in angiogenesis, glycolysis, and cell cycle control (25). HIF is a heterodimer comprising an O_2_-regulated subunit (HIF-1α or HIF-2α) and a constitutively expressed HIF-1β subunit (2–4). In normoxia, HIF-1α (and HIF-2α) protein is targeted for degradation through the hydroxylation of specific proline residues by a family of O_2_-dependent prolyl hydroxylase domain (PHD) enzymes (33, 51). At low O_2_ tension, PHD activity is inhibited, resulting in HIF-1 protein stabilization (12, 21, 32).

HIF-1α alters energy metabolism from mitochondrial oxidative phosphorylation to glycolysis by upregulation of glycolytic enzymes and inactivation of pyruvate dehydrogenase (27, 43, 62). It is assumed that less ROS are generated as a consequence of limited mitochondrial metabolism and shortage of oxygen, preserving the HSC reservoir from oxidative damage (23). Although detailed analysis of the role of HIF-1α in adult mouse HSCs first indicated that regulation of HIF-1α levels is critical for HSC maintenance in the hypoxic BM niche (57, 61), recent studies suggest that HIFs are dispensable for HSCs. In different conditional knockout mice that lacked either *Hif-1α*, *Hif-2α*, or both, or alternatively lack *Hif-1β* resulting in impaired HIF-1α and HIF-2α function, no evidence was provided for HSC effects (29, 65). Despite early studies demonstrating that *Hif-2α* knockout mice were embryonic lethal (46) or died some months after birth due to ROS-mediated multiorgan failure and metabolic abnormalities (52), constitutive or inducible loss of *Hif-2α* did not affect steady-state hematopoiesis, HSC numbers, or serial transplantation (14). Thus, evidence for HIF-mediated regulation of ROS in HSCs has yet to be provided.

In addition to HIFs, other oxygen-sensitive and hypoxia-responsive cellular pathways have been described that also might be involved in the protection of HSCs. Notably, a number of recent studies have shown that the transcription factor NF-κB, a critical regulator of innate immunity, inflammation, and apoptosis (63), is activated by hypoxia (4). In this study, we have investigated the effect of oxidative stress-induced cell death by DL-buthionine-(S,R)-sulfoximine (BSO) in HSCs and progenitor cells from mouse BM. BSO, a potent inhibitor of GSH biosynthesis that leads to an increase of intracellular ROS levels (13), has been previously used to induce oxidative stress in hematopoietic cells (20, 70, 71). Thus, we used BSO to experimentally mimic elevated levels of ROS *ex vivo* in FACS-sorted Lineage-Sca-1^+^c-kit^+^ (LSK) cells, a heterogeneous cell population enriched for primitive cells with self-renewal potential (41). High levels of GSH confer protection against oxidative stress whereas its depletion will challenge the cells with increased levels of ROS. We found that LSK cells cultured in hypoxia were protected from oxidative stress-induced cell death by BSO and that the *in vivo* repopulating ability of BSO-treated HSCs cultured in hypoxia but not normoxia was maintained. Importantly, no evidence was found for an involvement of HIF-1α or HIF-2α in the hypoxia-mediated protection. In contrast, NF-κB activity was identified as a putative component of hypoxia-induced protection to detrimental ROS effects.

## Results

### LT- and short-term-HSCs express lower levels of ROS than more committed progenitor cells

**FIG. 1.**
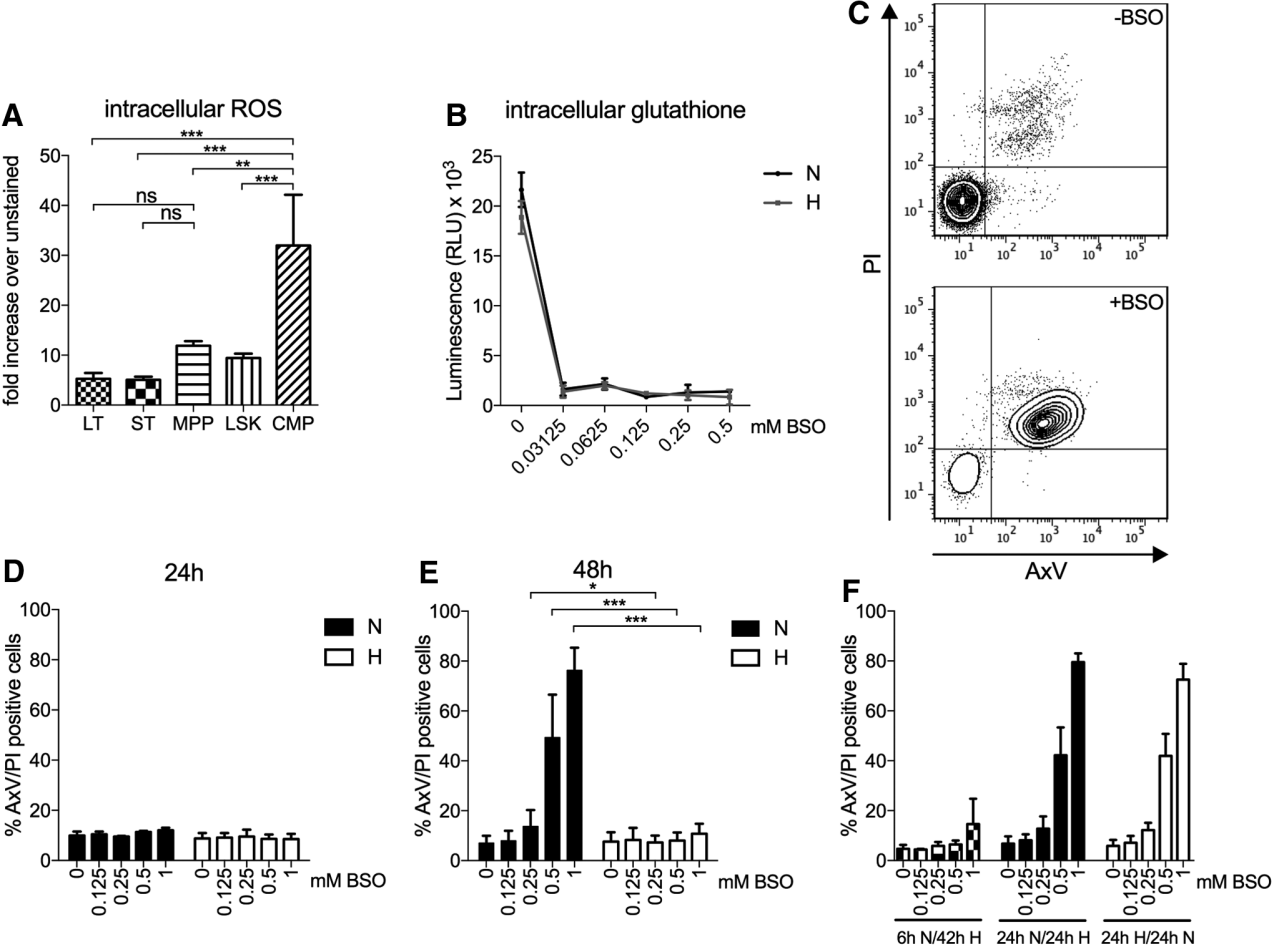
**Hypoxic treatment, but not preconditioning, protects LSK BM cells against BSO-mediated cytotoxicity. (A)** Flow cytometric evaluation of the intracellular level of ROS in H_2_DCFDA-stained LSK cells, LT-HSCs (CD34^−^Flt3^−^), ST-HSCs (CD34^+^Flt3^−^), MPPs (CD34^+^Flt3^+^), and CMPs (Sca-1^−^c-kit^+^CD16/32^int^ CD34^high^) normalized to PBS-treated cells (no H_2_DCFDA). Freshly prepared BM cells were enriched for c-kit expression, incubated with H_2_DCFDA, and finally analyzed by flow cytometry. Data are presented as mean ± SD (*n* = 3). Statistical analysis was performed by using Tukey's test. **(B)** Cells enriched for c-kit expression were cultured in H or N for 24 h with or without BSO (0.03125, 0.0625, 0.125, 0.25, and 0.5 mM), and the level of GSH × 10^3^ was measured by the GSH-Glo™ Glutathione luminescence-based assay kit. Data are presented as mean ± range (*n* = 2, in triplicates) in RLUs. **(C–F)** LSK cells were cultured in H or N with or without BSO (0.125, 0.25, and 0.5, 1 mM). **(C)** Representative flow cytometry data of cells stained for AxV and PI after 48 h of BSO treatment (0 and 1 mM) in N. **(D, E)** Flow cytometric evaluation of cell viability by AxV and PI staining of LSK cells cultured in H or N for 24 or 48 h. Data are presented as mean ± SD (*n* = 3–6). Statistical analysis was performed by using paired Student's *t*-test. **(F)** Flow cytometric evaluation of cell viability by AxV and PI staining of LSK cells cultured with BSO for 6 h N + 42 h H, 24 h N + 24 h H, or 24 h H + 24 h N. Data are presented as mean ± SD (*n* = 4–5). *p < 0.05, **p < 0.01, ***p < 0.001, and ns, nonsignificant. AxV, Annexin V; BM, bone marrow; BSO, DL-buthionine-(S,R)-sulfoximine; CMPs, common myeloid progenitors; GSH, glutathione; H, hypoxia; H_2_DCFDA, 6-carboxy-2′,7′-dichlorodihydrofluorescein diacetate; LSK, Lineage-Sca-1^+^c-kit^+^; LT-HSCs, long-term hematopoietic stem cell; MPPs, multipotent progenitors; N, normoxia; PBS, phosphate-buffered saline; PI, propidium iodide; RLUs, relative luminescence units; ROS, reactive oxygen species; SD, standard deviation; ST-HSCs, short-term hematopoietic stem cells.

Previous studies have shown that the LT engrafting ability of HSCs resides within the BM environment of low oxygen levels (44). However, the level of ROS in different hematopoietic populations has not been fully investigated. We, therefore, decided to stain populations from freshly isolated mouse BM with the intracellular ROS-indicator 6-carboxy-2′,7′-dichlorodihydrofluorescein diacetate (H_2_DCFDA), which is a chemically reduced, acetylated form of fluorescein used as an indicator for ROS in cells. The mean fluorescence signal for 2′,7′-dichlorofluorescein (DCF) staining (*i.e.*, ROS level) was significantly lower in LT- and short-term (ST)-HSCs compared with multipotent progenitors (MPPs) (2.3-fold increase compared with LT-HSCs) and common myeloid progenitors (CMPs) (6.1-fold higher than LT) (Fig. 1A), indicating that the earliest HSC compartment in the mouse BM displays lower levels of ROS.

### Hypoxia protects LSK BM cells from BSO-mediated cytotoxicity

Next, we investigated whether hypoxia affects the survival of HSCs when challenged with oxidative stress. To induce elevated levels of ROS, we used BSO, a selective irreversible inhibitor of γ-glutamylcysteine synthetase, which mainly decreases the level of cytoplasmic GSH, an intracellular antioxidant that plays a critical role in detoxifying ROS (34). BSO treatment of c-kit-enriched cells lead to a rapid decrease of the intracellular GSH level in cells cultured in both normoxia and hypoxia (Fig. 1B). The consequence of BSO treatment on viability was determined in cells cultured in normoxia and hypoxia for 24 or 48 h. One representative experiment for detection of apoptotic cells, as measured by Annexin V (AxV) binding and propidium iodide (PI) staining, is shown in Figure 1C. Although the viability after 24 h remained unchanged with few apoptotic cells detected (Fig. 1D), a dramatic induction of apoptosis of cells in normoxia with increasing concentrations of BSO was seen after 48 h (Fig. 1E). Apoptosis increased markedly with higher concentrations of BSO in normoxia [no BSO, 7.1% (±2.9); to 1 mM, 76.3% (±9.1)]. For cells cultured in hypoxia with increasing concentrations of BSO, cell death was as low as non-BSO-treated cultures [no BSO, 6.5% (±3.7); to 1 mM, 9.6% (±4.0)], demonstrating that hypoxia protects LSK cells from BSO-induced apoptosis.

To exclude any effects specifically caused by BSO itself, we determined to include another model of oxidative stress, paraquat (PQ), which induces oxidative stress by reacting with molecular oxygen in the mitochondria to generate superoxide (3). By the addition of PQ to LSK cells, we could show that even though PQ causes cell death in hypoxia, a significantly higher proportion of cells undergo apoptosis in normoxia under increasing concentrations of PQ (Supplementary Fig. S1). The difference in apoptosis was apparent at 0.25 mM PQ in normoxia [no PQ, 3.1% (±1.4%) to 0.25 mM, 81.7% (±6.7%)] compared with hypoxia [no PQ, 2.6% (±0.6%) to 0.25 mM, 39.8% (±3%)], mimicking the result seen with BSO and clearly demonstrating that hypoxia protects LSK cells from oxidative stress-induced cell death.

### Cells cultured in normoxia for 6 h still show protective effect against apoptosis when exposed to BSO in hypoxia

To address whether pre-conditioning would influence cells to BSO treatment, LSK cells were incubated in normoxia with BSO for 6 or 24 h and then moved to hypoxia for an additional 42 or 24 h, respectively. A 6-h exposure to normoxia and BSO was not enough to cause BSO-induced apoptosis in LSK cells after hypoxic culture, whereas 24 h was sufficient to affect the apoptotic process that could not be repressed by hypoxia (Fig. 1F). Thus, apoptosis increased with increasing doses of BSO when cells were incubated 24 h in normoxia followed by 24 h of hypoxia [no BSO, 9.9% (±1.6); to 1 mM, 65.5% (±23.9)]. When LSK cells were exposed to hypoxia and BSO for 6–24 h before normoxia for an additional 24 h, the pre-conditioning of hypoxia was not able to protect cells from BSO-induced cell death [no BSO, 6.9% (±3.6); to 1 mM, 72.8% (±10.7)]. Thus, 6-h pre-exposure to normoxia did not ablate the protective effects seen in hypoxia when exposed to BSO treatment. In addition, 24-h pre-exposure to hypoxia does not induce a protective molecular state to damaging effects by BSO-induced intracellular ROS that persists when cells are shifted to normoxic conditions.

### GSH-depleted cells in hypoxic culture maintain LT *in vivo* repopulation ability

**FIG. 2.**
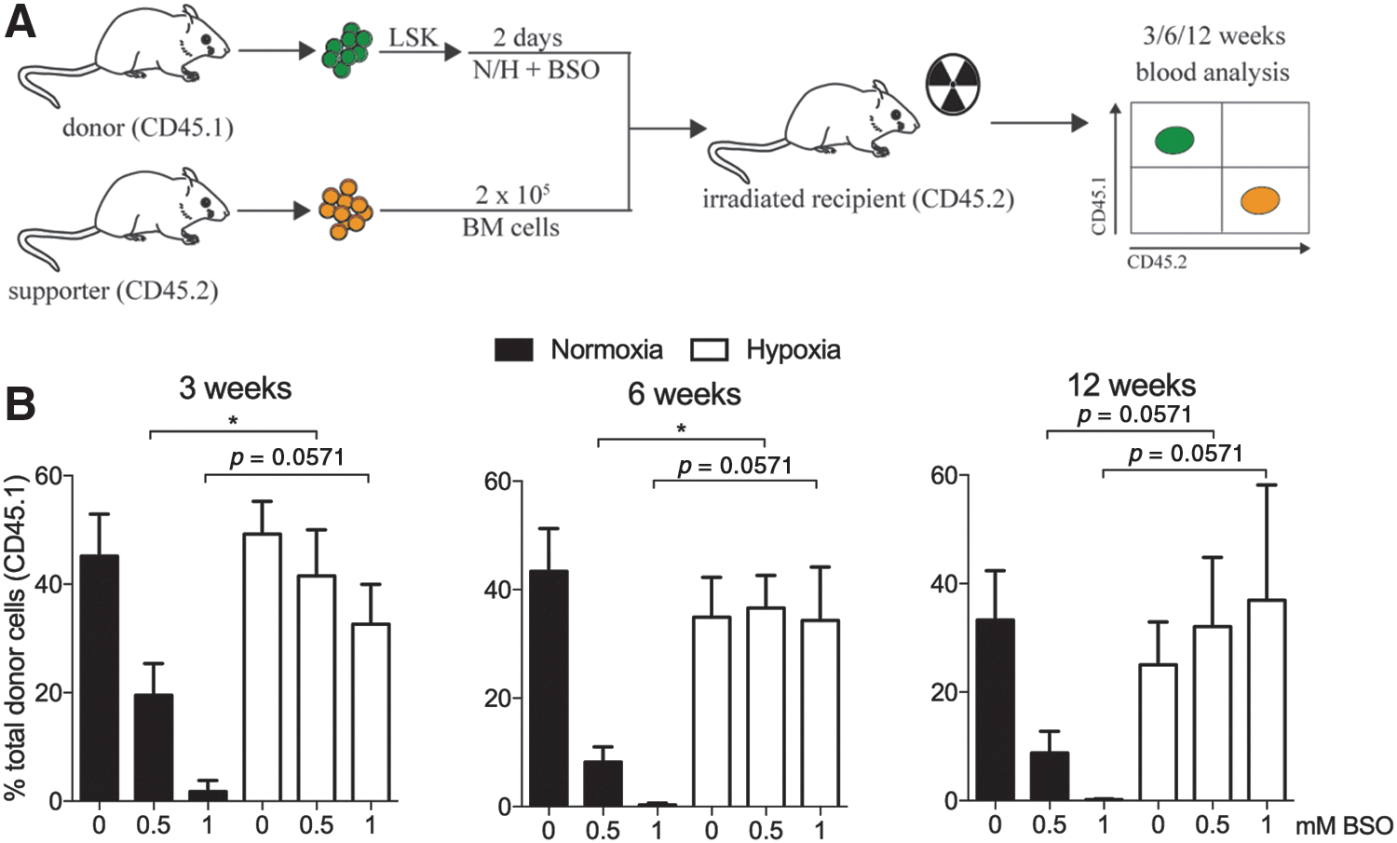
**Hypoxia maintains the repopulation ability of HSPCs under oxidative stress.** Noncompetitive repopulation assay with LSK cells cultured with increasing BSO concentrations for 48 h in H or N. **(A)** Schematic of non-competitive BM transplantation. Cultured cells equivalent to 2000 initially plated LSK cells (CD45.1) along with 2 × 10^5^ BM supporter cells (CD45.2) were transplanted into lethally irradiated (9 Gy) CD45.2 recipient mice. Donor and supporter cells were distinguished based on their expression of CD45.1 and CD45.2, respectively, which can be detected by flow cytometry. **(B)** Flow cytometric analysis of the frequency of donor-derived (CD45.1) cells in the peripheral blood of CD45.2-recipient mice at 3, 6, and 12 weeks after BM transplantation. Data are presented as mean ± SD (*n* = 3–4/group). Statistical analysis was performed by using Mann–Whitney *U*-test. *p < 0.05. HSPCs, hematopoietic stem cell and progenitor cells.

We next addressed whether hypoxic pre-conditioning protects the engrafting potential of hematopoietic stem and progenitor cells (here collectively called HSPCs) from detrimental effects by ROS. To distinguish donor cells from supporter cells, mice with allelic variants of the cell surface marker CD45 were used. Freshly isolated LSK cells from B6.SJL mice (CD45.1) were cultured for 2 days in normoxia or hypoxia with two concentrations of BSO (0.5 mM and 1 mM, respectively) before lateral tail vein injections into lethally irradiated C57BL/6J (CD45.2) recipients together with supporter cells (CD45.2) (Fig. 2A). The engraftment potential after 3 weeks was markedly decreased in mice that received cells treated with BSO under normoxic conditions (Fig. 2B, left). In contrast, donor cells cultured in hypoxia revealed a significantly higher repopulation of peripheral blood, indicating that hypoxia maintains the reconstitution capacity of HPSCs under *ex vivo* conditions of GSH depletion. Although a slight decrease in the repopulating ability 3 weeks post-transplantation of donor cells cultured in hypoxia with 1 mM BSO was seen, LSK cells reconstituted to a similar level with or without BSO treatment after 6 weeks (Fig. 2B, middle). LT engraftment measured at 12 weeks post-transplantation showed that it maintained the engraftment potential of cells cultured in hypoxia (Fig. 2B, right) and no difference in lineage distribution was detected in mice transplanted with HSPCs cultured in either hypoxia or normoxia (Supplementary Fig. S2). These data show that HSPCs in hypoxia challenged with depleted GSH and exposure to oxidative stress maintain the repopulative potential *in vivo.*

### Hypoxia protects LSK cells from BSO-induced production of mitochondrial superoxide

**FIG. 3.**
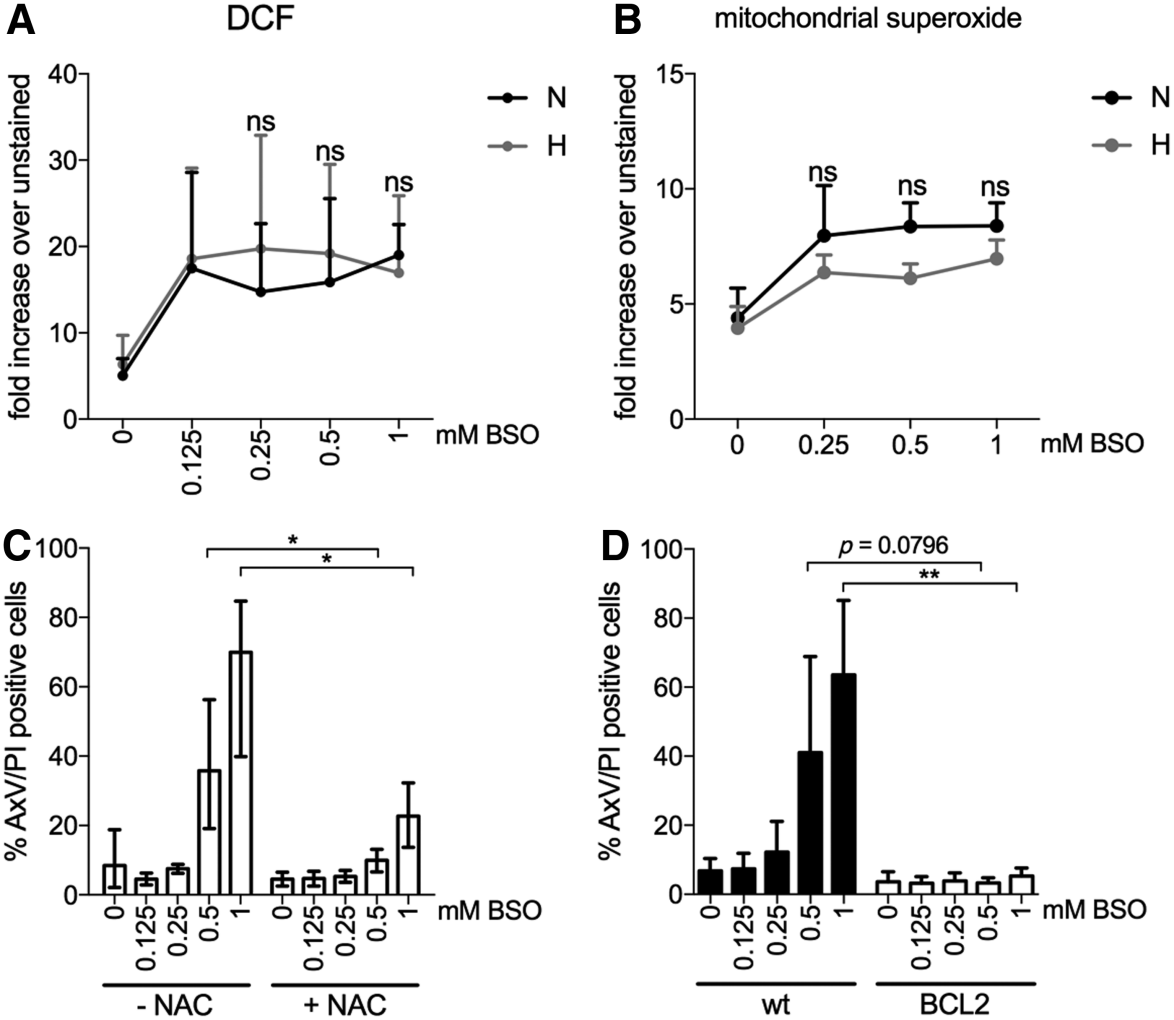
**Antioxidant NAC and BCL-2 protects LSK cells from BSO-induced cell death in N. (A)** Flow cytometric evaluation of the intracellular level of ROS in H_2_DCFDA-stained LSK cells cultured in H or N for 48 h with or without BSO (0.125, 0.25, 0.5, and 1 mM), normalized to PBS-treated cells (no H_2_DCFDA). Data are presented as mean ± SD (*n* = 3–4). Statistical analysis was performed by using a Wilcoxon signed-rank test. **(B)** Flow cytometric evaluation of mitochondrial superoxide ROS in MitoSOX Red-treated LSK cells cultured in H or N for 48 h with or without BSO (0.25, 0.5, and 1 mM), normalized to PBS-treated cells (no MitoSOX). Data are presented as mean ± SD (*n* = 3). Statistical analysis was performed by using paired Student's *t*-test. **(C)** Flow cytometric evaluation of cell viability by AxV and PI staining of LSK cells cultured in N for 48 h with or without BSO (0.125, 0.25, 0.5, and 1 mM) or NAC (1 mM). Data are presented as mean ± range (*n* = 2–4). Statistical analysis was performed by using paired Student's *t*-test. **(D)** Flow cytometric evaluation of cell viability by AxV and PI staining of LSK cells from C57BL/6J wt or C57BL/6J-*vav-bcl-2* mice cultured in N for 48 h with or without BSO (0.125, 0.25, 0.5, and 1 mM). Data are presented as mean ± SD (*n* = 3). Statistical analysis was performed by using unpaired Student's *t*-test. *p < 0.05, **p < 0.01, and ns, nonsignificant. DCF, 2′,7′-dichlorofluorescein; NAC, N-acetyl-L-cysteine.

To explore the role of ROS in the cytotoxicity of BSO and the ability of hypoxia to protect against apoptosis, we monitored the accumulation of ROS by staining LSK cells with the intracellular ROS-indicator H_2_DCFDA. The fluorescent intensity of oxidized DCF increased in both normoxic and hypoxic cultures with increasing BSO concentrations, indicating that a lower level of intracellular ROS is not responsible for reduced cell death by hypoxia (Fig. 3A). Intracellular ROS are generated endogenously *via* cellular signaling and redox reactions as well as by oxidative phosphorylation in mitochondria. In addition, different species of ROS such as the O_2_ itself, ^•^O_2_^−^, H_2_O_2_, and ^•^OH accommodate the effects seen by ROS (6, 39). Even though H_2_DCFDA is used extensively to measure intracellular ROS, it determines mainly the levels of H_2_O_2_, hydroxyl, and peroxyl radicals and excludes highly reactive mitochondrial-generated superoxide. We, therefore, decided to determine the effects of mitochondrial superoxide by MitoSOX in normoxic and hypoxic cells challenged by BSO treatment. As measured by flow cytometry, the levels of superoxide were elevated in cells in normoxia with increasing concentrations of BSO in comparison to hypoxia (Fig. 3B). Although the results are not significant, they imply reduced mitochondrial-generated superoxide accounting for hypoxia-mediated protection of apoptosis in BSO-treated cells. Further, as positive and negative controls, LSK cells treated with H_2_O_2_ or the ROS scavengers Tiron (15) and N-acetyl-L-cysteine (NAC) were included, which induce or reduce ROS, respectively (Supplementary Fig. S3).

### Antioxidants NAC and BCL-2 protect LSK cells from BSO-induced cell death in normoxia

Next, LSK cells were treated with the precursor of GSH, NAC, during 48-h exposure to increasing concentrations of BSO in normoxia. As seen in Figure 3C, NAC addition inhibited BSO-mediated apoptosis [no NAC: 0.5 mM BSO, 35.8% (±15.9); 1 mM BSO, 69.9% (±20.8); 1 mM NAC: 0.5 mM BSO, 10.0% (±3.5); 1 mM BSO, 22.7% (±9.5)]. Since the mitochondria are involved in apoptosis *via* the release of cytochrome c, we determined whether overexpression of anti-apoptotic BCL-2 could block the BSO effects. We sorted LSK cells from *Bcl-2* transgenic mice, overexpressing the BCL-2 protein in hematopoietic cells (40), and repeated the experiment with BSO treatment in normoxia. The presence of BCL-2 completely inhibited BSO treatment of LSK cells in normoxia (Fig. 3D). For instance, at 1 mM BSO, apoptosis remained at baseline level in LSK cells from *Bcl-2* mice [no BSO, 3.6% (±2.9); 1 mM BSO, 5.3% (±2.4)], whereas the majority of LSK cells from wild-type mice died by apoptosis [no BSO, 6.8% (±3.6); 1 mM BSO, 63.5% (±21.6)]. These data demonstrate that the effects by hypoxia during BSO treatment can be mimicked by adding NAC or using BCL-2-overexpressing cells in normoxia and that hypoxia protection against ROS assault needs mitochondrial involvement.

### HIF-1α is dispensable for hypoxia-mediated protection to oxidative stress-induced cell death

**FIG. 4.**
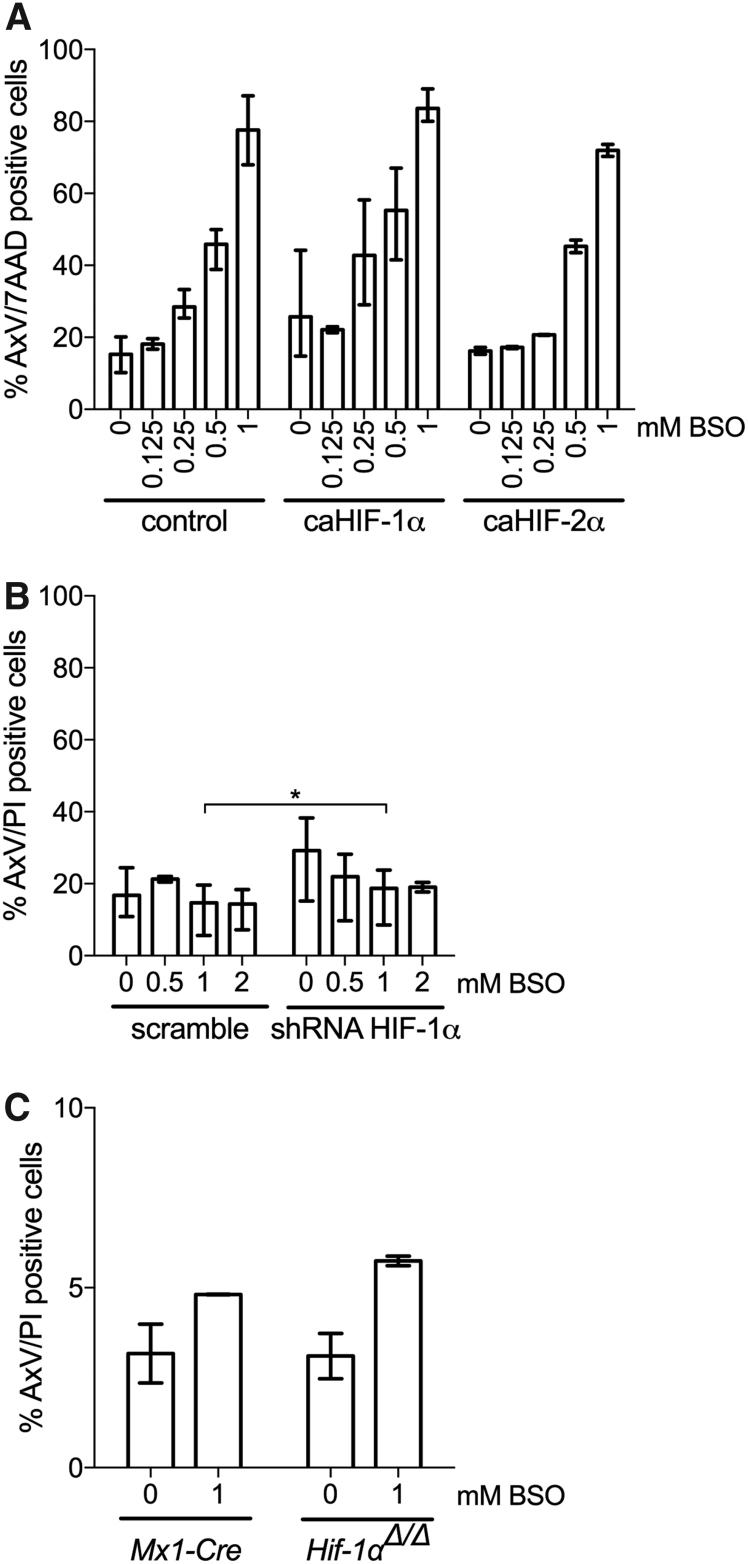
**HIF-1α is dispensable for H-mediated protection to oxidative stress-induced cell death. (A)** Flow cytometric evaluation of cell viability by AxV and 7AAD staining of LSK cells transduced with GFP vector (control), caHIF-1α or caHIF-2α cultured in N for 48 h with or without BSO (0.125, 0.25, 0.5, and 1 mM). LSK cells were transduced 24 h after culture in N, and they were sorted for GFP expression 2 days after transduction. Data are presented as mean ± range (*n* = 2–3). Statistical analysis was performed by using Dunnett's test to control shRNA. **(B)** Flow cytometric evaluation of cell viability by AxV and PI staining of LSK cells transduced with non-targeting shRNA-scramble control or shRNA-HIF-1α, cultured in H for 48 h with or without BSO (0.5, 1, and 2 mM). LSK cells were transduced 24 h after culture in H, and they were sorted for GFP expression 2 days after transduction. Data are presented as mean ± range (*n* = 2–3). Statistical analysis was performed by using paired Student's *t*-test. **(C)** Flow cytometric evaluation of cell viability by AxV and PI staining of LSK cells from pIpC-treated Hif-1α^+/+^ (*Mx1-Cre*) or *Hif-1α^Δ/Δ^* mice cultured in H for 48 h with or without BSO (1 mM). Data are presented as mean ± range (*n* = 2). *p < 0.05. 7AAD, 7-Aminoactinomycin D; GFP, green fluorescent protein; HIF, hypoxia-inducible factor; pIpC, polyI:polyC; shRNA, short hairpin RNA.

We next sought to determine the molecular pathway by which hypoxia preserves the integrity of HSPCs on GSH depletion and increased mitochondrial ROS. Since HIFs constitute the primary molecular response to hypoxia *via* protein stabilization on oxygen deprivation, we investigated whether LSK cells exposed to BSO in normoxia were protected by constitutively active, oxygen-insensitive versions of HIF. Due to point mutations of two critical proline residues, these mutated HIF proteins are not degraded when exposed to oxygen (9). LSK cells were retrovirally infected with vectors containing green fluorescent protein (GFP) and either HIF-1α (caHIF-1α) or HIF-2α (caHIF-2α). This leads to overexpression of both RNA and protein (Supplementary Fig. S4A–C and Fig. S10) (9). As a control, an empty GFP-containing retrovirus was used. Forty-eight hours after infection, LSK cells were FACS-sorted for GFP expression and next cultured in normoxia and increasing BSO concentrations. No significant difference in the numbers of apoptotic cells was seen between control cells and cells transduced with HIF-1α or HIF-2α [control, 77.7% (±9.6); caHIF-1α, 83.7% (±4.8); caHIF-2α, 71.9% (±2.4) at 1 mM BSO)] (Fig. 4A), demonstrating that overexpression of HIF is not sufficient to protect against ROS.

We also tested whether knockdown of *Hif-1α* was able to increase apoptosis induced by BSO in hypoxia. To this end, lentiviral short hairpin RNAs (shRNAs) to *Hif-1α* previously utilized in our studies (16) and displaying suppression of *Hif-1α* expression (Supplementary Fig. S5) were used to infect LSK cells with GFP- and shRNA-containing vectors. Similar to the results with cells transduced with scramble shRNA and exposed to BSO [16.8% (±7.0) for no BSO; 17.9% (±0.6) at 2 mM BSO], the numbers of apoptotic cells after knockdown of *Hif-1α* made no significant difference [2 mM BSO, 19.0% (±1.9)] (Fig. 4B).

To exclude any *in vitro* effects of manipulating HIF expression, we also determined the functional effect of HIF-1α depletion by analyzing an inducible *Hif-1α* deletion model *in vivo* (50). To delete the *Hif-1α* gene in HSCs, we induced a Cre transgene in *Mx1-Cre:Hif-1α^flox/flox^* mice by sequential polyI:polyC (pIpC) injections, which efficiently excised the *Hif-1α* gene in BM cells (Supplementary Fig. S6). We then isolated LSK cells from the BM of three successfully treated mice and four treated *Mx1-Cre* control mice (lacking floxed *Hif-1α*) and cultured them for 48 h with 1 mM BSO in hypoxia. Importantly, *Hif-1α* gene ablation did not result in any major increase in apoptosis compared with control LSK cells cultured in hypoxia with 1 mM BSO [control, 4.8% (±0.0); *Hif-1α^Δ/Δ^*: 5.7% (±0.2)] (Fig. 4C). Collectively, these results suggest that HIF-1α is dispensable for hypoxia-induced protection to ROS effects on GSH depletion.

### Gene expression profile of hypoxic LSK cells

**FIG. 5.**
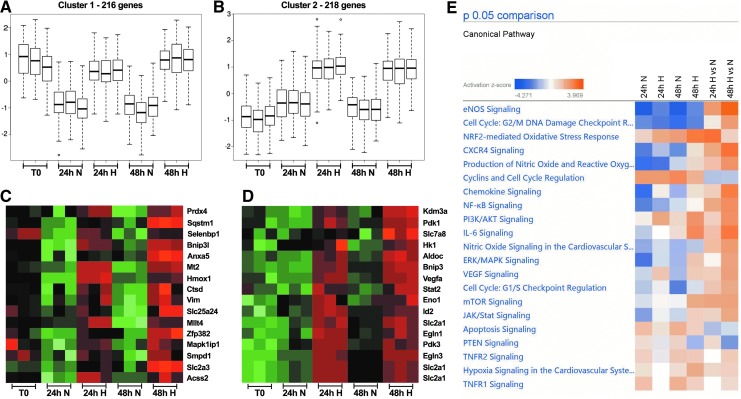
**Gene expression and pathway analysis of LSK cells reveals potential involvement of the NF-κB pathway in H. (A, B)** Box plots resolving genes into two clusters (three replicates) as either maintained **(A)** or upregulated **(B)** by H. The *whiskers* represent the total expression range of the sample within each cluster, *horizontal bars* represent the median expression, and *boxes* represent the 75th and 25th percentile of the data. **(C)** Heat map showing the relative expression of a selection of genes maintained by H. **(D)** Heat map showing the relative expression of a selection of genes upregulated by H. Both maps are based on microarray analysis from two independent experiments performed in triplicates (replicates averaged, *green* to *red* denotes low to high expression). **(E)** Ingenuity Pathway Analysis of canonical pathways of data sets from microarray of LSK cells treated in N or H for 24 and 48 h, respectively. *Red* colored genes are overexpressed as compared with time point 0 h of freshly sorted LSK cells; *blue* are underexpressed (*p* < 0.05). NF-κB, nuclear factor-kappa B.

To identify pathways that are important for LSK cells in hypoxia, we determined the transcriptional profile by using microarray analysis. Two batches of LSK cells were sorted and cultured in triplicates in hypoxia or normoxia with stem cell factor (SCF), thrombopoietin (TPO), and interleukin (IL)-6 for 24 or 48 h, respectively. One-third of the freshly sorted LSK cells were set aside to prepare RNA as control (T0). Equal amounts of RNA from the three time points (0, 24, and 48 h) were isolated and independently hybridized to Affymetrix GeneChip^®^ Mouse Gene 2.0 ST, obtaining three biological replicates for each experimental condition. Differentially expressed genes were partitioned into several clusters corresponding to differential expression between freshly sorted LSK cells and cells exposed to normoxia or hypoxia for 24 or 48 h. The analysis was focused on two subsets of regulated genes: those preserved in hypoxia but downregulated in normoxia (cluster 1; Fig. 5A) and those that were induced by hypoxia for both 24 and 48 h (cluster 2; Fig. 5B). Pairwise comparison among the samples from normoxic and hypoxic samples revealed differential expression of a large number of transcripts.

As the antioxidant enzymes CAT and SOD are known to be involved in reducing the levels of ROS, and previously have been indicated to be target genes for HIF-2α (35, 52), we first determined whether the expression of *Cat* and *Sod* was upregulated in hypoxia. The expression levels were analyzed by quantitative real-time PCR (qRT-PCR) as well as by a more targeted analysis of the microarray analysis. However, neither *Cat* nor *Sod* expression was upregulated by hypoxia (Supplementary Fig. S7), suggesting that HIF-2α is not involved in ROS protection by hypoxia.

The microarray analysis identified 216 genes maintained and 218 genes upregulated by hypoxia. Several genes belonging either to oxidative stress, including heme oxygenase 1 (*Hmox1*), or to the apoptotic or the autophagy pathway, such as *Bnip3I* and *Sqstm1*, were maintained in hypoxia (Fig. 5C). Interestingly, the expression of *Prdx4* belonging to the peroxiredoxin family was also maintained in hypoxia. This gene encodes a protein that acts as an antioxidant (69), and it has been found to play a regulatory role in the activation of the transcription factor NF-κB (24). The expression of Prdx4 by hypoxia was confirmed by qRT-PCR (Supplementary Fig. S8).

Several of the upregulated messenger RNAs have previously been identified as hypoxia-induced genes, such as *Egln1*, *Egln3*, *Eno1*, *Hk1*, *Kdm3a/Jmjd1*, *Pdk1*, *Pdk3*, *Slc2a1/Glut1*, and *Vegfa.* In addition, novel genes encoding proteins involved in glycolysis, metabolism and metabolite transport, or oxidative stress protection (for instance *Aldoc* and *Slc7a8*) were also upregulated by hypoxia (Fig. 5D). We could confirm the upregulation by hypoxia of some of these genes by qRT-PCR (*Hk1*, *Pdk1*, and *Vegfa*) (Supplementary Fig. S8).

Given the previously verified transcriptional upregulation of several cellular pathways in hypoxia and oxidative stress, including the transcription factors NF-κB and NRF2, we decided to perform Ingenuity Pathway Analysis (IPA) of cellular pathways to interpret the data on differentially expressed genes. The dataset containing gene identifiers and corresponding expression values were uploaded into the IPA software, and each gene identifier was mapped to its corresponding pathway. The analysis showed that several pathways involved in hypoxia and oxidative stress were upregulated during the 24- to 48-h incubation in hypoxia, of which eNOS, NRF2, CXCR4, and NF-κB signaling was among the most abundant (Fig. 5E).

### PHD inhibitor protects LSK cells from GSH depletion

**FIG. 6.**
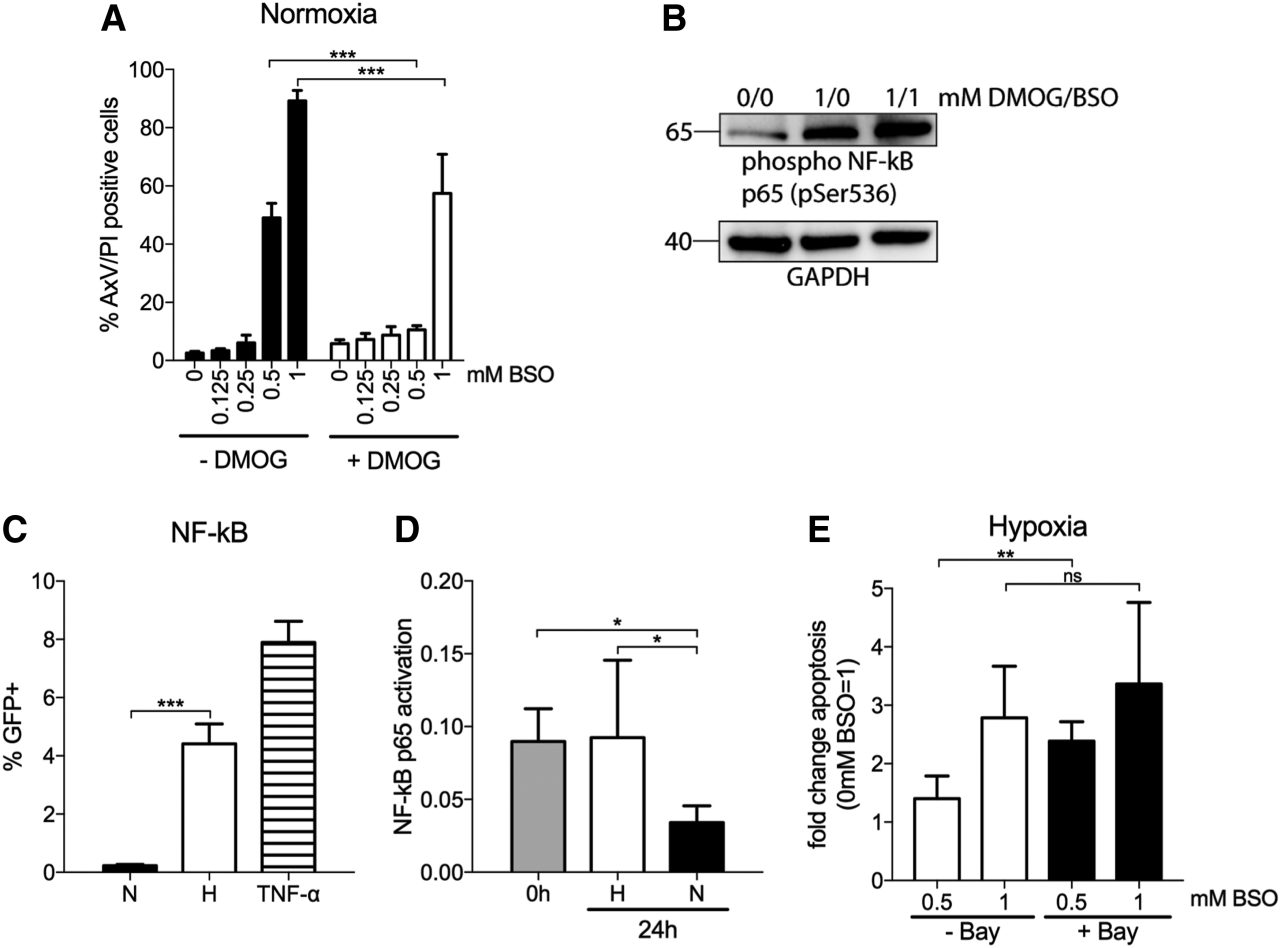
**Prolyl hydroxylase-treatment recapitulates H-induced protection to BSO *via* NF-κB activation. (A)** Flow cytometric evaluation of cell viability by AxV and PI staining of LSK cells cultured in N for 48 h with or without BSO (0.125, 0.25, 0.5, and 1 mM) or DMOG (125 μM). Data are presented as mean ± SD (*n* = 5). Statistical analysis was performed by using paired Student's *t*-test. **(B)** Cytoplasmic protein levels of p65 in c-kit^+^ cells treated with or without 1 mM DMOG and 1 mM BSO for 4 h in N were analyzed with Western blot. Loading control used was antibody against the house-keeping protein GAPDH. Full blots in Supplementary Figure S11. **(C)** Flow cytometric evaluation of LSK cells transduced with a lentivirus reporter construct containing three NF-κB consensus sites driving GFP expression. LSK cells were transduced 24 h after culture in N; 2 days post-transduction, cells were incubated in H or N for an additional 48 h and analyzed for GFP expression. As a positive control, 100 ng/mL tumor necrosis factor-α was added. Data are presented as mean ± SD (*n* = 5–7). Statistical analysis was performed by using paired Student's *t*-test. **(D)** Nuclear extracts from freshly isolated c-kit^+^ cells or c-kit^+^ cells incubated in H or N for 24 h were assayed by using TransAM™ for p65 NF-κB activity. Data are presented as mean ± SD (*n* = 4). Statistical analysis was performed by using Dunn's test to N. **(E)** Flow cytometric evaluation of cell viability by AxV and PI staining of LSK cells cultured in H for 48 h with BSO (0.5 and 1 mM) with or without Bay 11-7082 (5 μM) and normalized to the BSO-nontreated cells (0 mM BSO). Data are presented as mean ± SD (*n* = 5). Statistical analysis was performed by using paired Student's *t*-test. *p < 0.05, **p < 0.01, ***p < 0.001, and ns, nonsignificant. DMOG, dimethyloxalylglycine.

In the presence of oxygen, HIFs are ubiquitinated and degraded by proline hydroxylation executed by PHDs (53). Although HIFs are the best identified substrates (53), previous studies have demonstrated that some PHDs are involved in the degradation of NF-κB (37). To investigate whether the NF-κB pathway is involved in hypoxia protection to BSO, we first tested whether the PHD inhibitor dimethyloxalylglycine (DMOG), used to detect hypoxia-induced, but HIF-1α-independent effects (28, 60, 68), could mimic hypoxia-induced protection to BSO. LSK cells were incubated with increasing concentrations of BSO in the presence of DMOG (1 mM). As seen from Figure 6A, apoptosis in normoxia by 1 mM BSO was partially abrogated with DMOG [89.2% (±3.6) without DMOG *vs.* 55.1% (±13.3) with DMOG]. At 0.5 mM BSO, the effects by DMOG was even more profound [49.0% (±5.0) without DMOG *vs.* 10.6% (±1.3) with DMOG]. The result suggests that DMOG suppresses an oxygen-sensitive pathway that otherwise is degraded by proline hydroxylation in normoxia. To verify that DMOG induces activation of NF-κB and that the protective effect of hypoxia on BSO-induced cell death is regulated by NF-κB, we cultured c-kit^+^ cells in normoxia for 4 h with the addition of DMOG and BSO. Interestingly, we could show that phosphorylation of p65 (pSer536) is induced by DMOG, and it increased further with the addition of BSO (Fig. 6B; Supplementary Fig. S11).

### NF-κB activity is enhanced by hypoxia

Since the NF-κB pathway was identified in the IPA analysis and p65 was shown to be phosphorylated by DMOG and BSO, we decided to focus on NF-κB as one pathway activated by hypoxia in LSK cells and involved in ROS protection. NF-κB is a critical determinant of stress response to hypoxia and plays an important role in the control of apoptosis (42). The most abundant form is the 50-kDa (p50) and 65-kDa (p65 or RELA) subunits that are kept in the cytoplasm in an inactive state by binding to the inhibitor of κB (IκB) protein. In response to stimuli, IκB becomes phosphorylated, ubiquitinated, and degraded by the proteasome, resulting in the nuclear translocation of NF-κB and the activation of target genes (17, 22).

We investigated the effect of hypoxia on NF-κB activity by two experimental approaches, both methods measuring NF-κB transcriptional activity when binding to DNA targets. First, we transduced LSK cells with a lentiviral vector containing three consecutive NF-κB binding sites upstream of a GFP reporter gene. After 48 h of transduction without selection, cells were incubated for an additional 48 h in normoxia or hypoxia. Incubation in hypoxia resulted in a significant upregulation of GFP expression compared with cells in normoxia (19-fold, Fig. 6C), demonstrating NF-κB activation by hypoxia. The effect of hypoxia on NF-κB activity was also determined with the DNA-binding enzyme-linked immunosorbent assay (ELISA) measuring nuclear extracts of c-kit^+^ cells exposed to hypoxia or normoxia for 24 h. Although NF-κB activity was significantly abrogated in cells exposed to normoxia, it was maintained at the same level as in freshly isolated LSK cells on hypoxia (Fig. 6D). These results demonstrate that NF-κB signaling is maintained by hypoxia *in vitro*. This strengthens our results and suggests that NF-κB is active in the cells due to the hypoxic nature of the BM microenvironment.

### NF-κB activity is involved in protection to GSH depletion

To investigate whether the protective effect of hypoxia on BSO-induced cell death could be inferred, LSK cells were incubated in BSO together with the irreversible IκB-phosphorylation, Bay 11-7082, which prevents the release of p65 by hypoxia (47). Bay 11-7082 was able to partially abrogate hypoxia-mediated resistance to BSO treatment (Fig. 6E), and the number of apoptotic cells in hypoxia increased 3.4-fold when cells were co-incubated with 5 μM inhibitor together with 1 mM BSO compared with a 2.8-fold increase in hypoxic cells incubated only with BSO. Hence, NF-κB signaling appears to be a crucial component in HSPCs for hypoxia-induced protection to oxidative stress.

## Discussion

There is now evidence that HSCs have a low metabolic activity that spares them the damage of metabolic products such as ROS and DNA replication (20, 23). Since HSCs reside in a hypoxic BM microenvironment, ensuring their LT maintenance, it is assumed that hypoxia makes them more resistant to oxidative stress (57). Although HIF-1α and HIF-2α are expressed in HSCs and regulate their function and metabolic phenotype (7, 61, 62), it is unknown whether they are involved in the regulation of HSCs to oxidative stress and detrimental effects of ROS accumulation. The novel finding of this study is that mouse HSPCs are protected from oxidative stress by hypoxia in an HIF-independent manner.

We took a pharmacologic approach to increase ROS levels in LSK cells by inhibiting the rate-limiting enzyme in GSH biosynthesis with BSO, leading to depletion of GSH in the cells. GSH depletion is a hallmark of the progression of cell death and is often regarded as a marker of oxidative stress (11). Although LSK cells cultured in normoxic conditions succumbed to apoptotic cell death when challenged with BSO treatment, hypoxic conditions repressed the apoptotic effect and maintained the repopulation ability of transplantable HSPCs at high concentrations of BSO. Although ST engraftment (3 weeks) of LSK cells cultured in hypoxia was slightly decreased by BSO treatment, the LT effects after 6 or 12 weeks were conclusive for hypoxia to maintain a protective state. Importantly, the *in vivo* results show that stem and progenitor cells are protected from induced oxidative stress when cultured in hypoxic conditions compared with normoxic conditions. The results indicate that LT-HSCs are more resistant to oxidative stress when in hypoxia compared with more differentiated MPP cells. This is supportive to the concept that exposure to hypoxia in the BM microenvironment is essential to provide a sanctuary protecting HSCs from toxic damages and mutations acquired by oxidative stress.

A high level of O_2_ favors the formation of ROS (49), and studies have shown that in hypoxic cells the oxidative damage to cells is reduced (10, 31). Alternatively, the diminishing of ROS by hypoxia could be regulated by activation of pathways scavenging the cell of detrimental ROS by increased expression of specific genes. By limiting the amount of oxygen available, it is possible that less ROS are formed. Measurement of intracellular ROS demonstrated that LT- and ST-HSCs express lower levels of ROS compared with MPPs. However, since MPPs, in turn, expressed significantly lower levels than more differentiated CMPs, this suggests that the hypoxic nature of the BM regulates ROS at all different stages within the HSC compartment. These findings are to some extent in agreement with the findings that the intracellular levels of CMPs and GMPs correlate with the differentiation potential (55). Alternatively, these cells are capable of activating separate antioxidant redox systems. In experiments where BSO-treated LSK cells were pre-exposed to 24-h hypoxia before culture in normoxic conditions, cell death was not inhibited. Likewise, cells exposed to normoxia before hypoxic cultures could not be protected, indicating that cell damage is induced during the first 24 h of BSO treatment and that hypoxia is unable to inhibit this irreversible process.

When seeking the mechanisms by which hypoxia protects against BSO-induced cell death, we could demonstrate that both the antioxidant reagent NAC and expression of anti-apoptotic BCL-2 protein could rescue LSK cells challenged by GSH depletion. Thus, mitochondrial ROS seems to execute the detrimental effects by BSO in agreement with elevated levels of mitochondrial superoxide that were found in BSO-treated cells in normoxia. Utilizing oxygen-stable HIF-1α or HIF-2α as well as shRNA to *Hif-1α*, we could not link the hypoxia-induced protection of ROS to HIF function. Even with LSK cells from *Hif-1α* knockout mice, we were unable to demonstrate any role for HIF-1α in ROS regulation by hypoxia. Although we did not extend the analysis to include shRNA or knockout mice for *Hif-2α*, the expression of HIF-2α target antioxidant genes *Cat* and *Sod* when determined by qRT-PCR and microarray analysis were not upregulated in hypoxia. Together, these results indicate that oxygen-sensitive pathways independent of HIFs appear to be essential to execute the protective effects against an increase in ROS.

HIF activity is mainly regulated post-translationally at the protein level by PHD whose α-subunits are rapidly degraded under normoxia. When testing whether PHDs are involved in the hypoxia-mediated protection of HSCs from oxidative stress, we could show that LSK cells were profoundly rescued from apoptosis by BSO treatment in normoxia with DMOG, a pan-PHD inhibitor, suggesting that oxygen-sensitive signaling pathways are involved.

Besides the major role of HIFs in executing hypoxia-mediated effects in different cell types, multiple regulatory pathways have been shown to be controlled by hypoxia and could possibly be involved in the protection to elevated levels of ROS in HSCs and progenitor cells. A recent study identified several genes upregulated by hypoxia, but not by HIF-1α or HIF-2α (67), including *Hmox1*, which has been shown to be regulated by NRF2 (1). NRF2 has been shown to be involved in the regulation of oxidative stress, and since the recent study indicated that hypoxia-induced expression of heme oxygenase 1 (HMOX1) is repressed when NRF2 is downregulated, this pathway, and not HIFs, could be involved in the hypoxic response seen herein. However, we could not identify the NRF2 pathway as one of the pathways regulated in LSK cells by hypoxia, as it was only transiently affected by hypoxia and *Hmox1* was maintained in LSK cells after 24 h of hypoxic exposure but decreased in expression after 48 h.

We found several other potential target genes involved in metabolism and cell survival that were either upregulated or maintained in LSK cells after *ex vivo* culture by hypoxia.

By computational pathway analysis, we identified the NF-κB pathway to be upregulated by hypoxia. NF-κB has been shown to be a critical determinant of stress response to hypoxia (42). In response to hypoxia, the canonical NF-κB pathway is activated by the same PHDs that confer oxygen sensitivity to the HIF pathway (5). Accordingly, IκB becomes phosphorylated, ubiquitinated, and degraded by the proteasome, resulting in the liberation and nuclear translocation of NF-κB followed by binding to DNA consensus sites and activation of target genes. By different approaches, we could demonstrate augmented NF-κB activity in LSK cells exposed to hypoxia. NF-κB appears to be important also at steady state for HSPCs located in the hypoxic BM since freshly isolated LSK cells displayed as high NF-κB activity as LSK cells exposed to hypoxia *in vitro*. When hematopoietic progenitor cells from mouse BM were treated for 4 h with DMOG, NF-κB activity increased as measured by phosphorylation of serine 536. Previous studies have implied that GSH depletion by BSO treatment can affect NF-κB activity (26), raising the possibility that BSO and hypoxia may converge on the same pathway. Interestingly, the addition of BSO further enhanced this phosphorylation, suggesting that although detrimental to cells in normoxia, BSO treatment could affect NF-κB activity in cells in hypoxia.

Collectively, our study contributes to the notion that although HIF-1α is involved in metabolic adaption of HSCs by shifting energy production to glycolysis and away from mitochondrial respiration, HIF-1α (and HIF-2α) is not essential for the protection to harmful ROS. Our study indicates that NF-κB activity is a putative component of hypoxia-induced protection to detrimental ROS effects. The results are also supportive to the hypothesis that exposure to hypoxia in the BM microenvironment is essential to provide a sanctuary protecting HSCs from toxic damages and mutations acquired by oxidative stress.

## Materials and Methods

### Mice

Mice were bred and maintained in the animal facility at Linköping University. The Animal ethics committee at Linköping University approved all mouse experiments. Offspring were genotyped by PCR-based assay with DNA from mouse ear snips. B6.SJL (CD45.1) mice that express Ly5.1 and C57BL/6J (CD45.2) mice that express Ly5.2 were used as donors and recipients for experiments. C57BL/6J-*vav-bcl-2* (*Bcl-2*) transgenic mice were provided by G. Nilsson (Karolinska Institutet, Stockholm, Sweden) (40). *Hif-1α^Δ/Δ^* mice were provided by Dr. J. Cammenga (Linköping, Sweden) (50). *Hif-1α^flox/flox^* (B6.129-HIF1a^tm3Rsjo^/J; Jackson Laboratory, Bar Harbor, ME) mice were mated to *Mx1-Cre* mice to generate *Mx1-Cre:Hif-1α^flox/flox^* mice. Generation of *Hif-1α^Δ/Δ^* mice was induced by an intraperitoneal injection of 400 μg of pIpC (P1530; Sigma-Aldrich, St Louis, MO) on 3 consecutive days, into 7–12-week-old mice. As a control, age-matched pIpC-treated *Mx1-Cre* mice were used to outrule any side effects by pIpC treatment. Four weeks after the last injection of pIpC, mice were checked regarding gene deletion by PCR and used in experiments. The following primers were used in PCR amplification and sequencing reactions:

Human Bcl-2-forward: 5′-TGAGTACCTGAACCGGCACCT-3′
Human Bcl-2-reverse: 5′-ACCAGGGCCAAACAGAGCAGA-3′
Mx1-Cre-forward: 5′-CATGTGTCTTGGTGGGCTGAG-3′
Mx1-Cre-reverse: 5′-CGCATAACCAGTGAAACAGCAT-3′
Hif-1α^flox/flox^-forward: 5′-CGTGTGAGAAAACTTCTGGATG-3′
Hif-1α^flox/flox^-reverse: 5′-AAAAGTATTGTGTTGGGGCAGT-3′
Hif-1α^Δ/Δ^-forward: 5′-GCAGTTAAGAGCACTAGTTG-3′
Hif-1α^Δ/Δ^-reverse: 5′-TTGGGGATGAAAACATCTGC-3′
Hif-2α-forward: 5′-AGCTCGGAGAGGAGGAAGGA-3′
Hif-2α-reverse: 5′-TCCTTCCTCCTCTCCGAGC-3′

### Flow cytometry and antibodies

BM cells were harvested by crushing femurs, tibiae, and ilium from 8- to 12-week-old mice and enriched by positive selection for c-kit expressing cells using immunomagnetic beads (130-091-224; Miltenyi Biotec, Bergisch Gladbach, Germany). The enriched cells were incubated with CD16/32 (Fc)-block (553142, 2.4G2; BD Biosciences, San Jose, CA) (not CMP). For isolation of LSK cells, APC- or FITC-conjugated CD117 (c-kit) (105812 and 105806, 2B8) and FITC- or Pacific Blue™-conjugated Sca-1 (108105 and 108119, D7) antibodies were added. For isolation of CD34^−^Flt3^−^ (LT-HSC), CD34^+^Flt3^−^ (ST-HSC), and CD34^+^Flt3^+^ (MPP) cells (56), FLT3-Biotin (135307, A2F10), streptavidin-Pe-Cy7 (405206), and CD34-PE (119307, MEC14.7) antibodies were added. For isolation of Sca-1^−^c-kit^+^CD16/32^int^ CD34^high^ (CMP cells), CD16/32-APC (17-0161-81, 93; eBioscience, San Diego, CA) was added. Pe-Cy5-conjugated antibodies were used against lineage markers Gr1 (108410, RB6-8C5), Mac-1 (101210, M1/70), B220 (103210, RA-3-6B2), CD3 (100310, 145-2c11), and Ter119 (116210). All antibodies were purchased from BioLegend (San Diego, CA) unless stated otherwise. Dead cells were detected with PI (1 μg/mL, P3566; Molecular Probes, Eugene, OR). Labeled cells were sorted on an FACSAria™ (BD Biosciences). The purity of sorted LSK cells was always checked by reanalysis and it showed purity above 96% (Supplementary Fig. S9).

### Cell culture and hypoxic treatment

LSK cells were cultured in StemSpan serum-free medium (09600; Stem Cell Technologies, Vancouver, BC) with 50 ng/mL of murine SCF (250-03), human TPO (300-18), and human IL-6 (200-06), and c-kit^+^ cells were cultured with the addition of 50 ng/mL human FLT3-ligand (300-19) (all from PeproTech, Inc., Rocky Hill, NJ). Standard conditions for normoxia (20% O_2_) were 37°C in 5% carbon dioxide (CO_2_), whereas hypoxia (1% O_2_) was reached by incubation in a CO_2_/O_2_ incubator where an injection of N_2_ displaces atmospheric oxygen to the desired oxygen level detected by a sensor (Innova CO-14 incubator; New Brunswick Scientific CO, Edison, NJ). FDCP1 cells were cultured in Iscove's Modified Dulbecco's Medium (BE12-726F; Lonza, Verviers, Belgium) supplemented with 10% heat-inactivated fetal bovine serum (SV30160.03, HyClone; Thermo Fisher Scientific, Göteborg, Sweden), 2 mM L-glutamine (M11-004; PAA Laboratories, Pasching, Austria), and 5% IL-3 supernatant. Standard conditions for culture were 37°C in 5% CO_2_.

### Treatments

LSK cells were cultured with 1 mM NAC (A8199), Tiron (4,5-dihydroxy-1,3-benzenedisulfonic acid disodium salt monohydrate, 0.1–1 mM, 172553), 125 μM DMOG (D3695), or 5 μM Bay 11-7082 inhibitor (B5556) together with BSO (125–2000 μM, B2640) or PQ (125–500 μM, 856177) (all from Sigma-Aldrich) for the time indicated. As a positive control, LSK cells were treated for 1 h with H_2_O_2_ solution (200–300 μM, H1009; Sigma-Aldrich).

### Cell viability analysis

Cells were harvested, washed once with cold phosphate-buffered saline (PBS), and stained with FITC- or Alexa Fluor 647-conjugated AxV (640906 and 640912; BioLegend) and PI (2.5 μg/mL; Molecular Probes) or 7-Aminoactinomycin D (7AAD) (5 μg/mL, A9400; Sigma-Aldrich). In apoptotic cells, the membrane phospholipid phosphatidylserine translocates from the inner to the outer side of the plasma membrane, to which flurochrome-conjugated AxV with its high affinity to phospholipids can bind and detect apoptosis. By the addition of PI, which can enter the cell on loss of membrane integrity, early and late apoptosis can be distinguished. After incubation for 15 min at room temperature, cells were analyzed by flow cytometry on FACSCanto™ II (BD Biosciences).

### Flow cytometry analysis of intracellular ROS by H_2_DCFDA staining

To measure intracellular levels of ROS, cells were stained with 10 μM H_2_DCFDA (C400; Molecular Probes) for 30 min at 37°C and then washed with PBS. H_2_DCFDA is a chemically reduced, acetylated form of fluorescein used as an indicator for ROS in cells. It is converted to a highly fluorescent form, DCF, when the acetate groups are removed and oxidation by ROS activation occurs. Dead cells were detected with 7AAD (0.5 μg/mL). DCF fluorescence was detected by flow cytometry in the FL1 channel, and the mean fluorescence intensity (MFI) of stained cells was compared with that of unstained control cells.

### Flow cytometry analysis of mitochondrial superoxide by MitoSOX

To measure mitochondrial levels of superoxide, cells were stained with 2 μM MitoSOX Red (M36008; Molecular Probes) for 30 min at 37°C, washed with Hank's balanced salt solution, and finally detected by flow cytometry. MitoSOX permeates the cell membrane of live cells, targets the mitochondria, and on oxidation by superoxide exhibits fluorescence. The MFI of stained cells was compared with that of unstained control cells.

### GSH measurement

The level of GSH was studied with the GSH-Glo™ Glutathione Assay Kit (V6911; Promega, Madison, WI), as recommended by the manufacturer. Briefly, c-kit^+^ cells were harvested, washed once with PBS, and suspended in PBS. Two times GSH-Glo reagent was prepared immediately before use and added to each sample for 30 min at room temperature. Luciferin detection reagent was added to each sample for 15 min at room temperature, and the luminescence generated was measured with a VICTOR3 multilabel reader (PerkinElmer, Inc., Waltham, MA).

### Lentiviral and retroviral vectors

Complementary DNAs (cDNAs) encoding murine *Hif-1α* (caHIF-1α; double mutant P402A/P563A) or murine *Hif-2α* (caHIF-2α; double mutant P405A/P531A), both carrying two-point mutations rendering the proteins insensitive to oxygen-dependent degradation, were cloned in the retroviral vector pMy-EGFP (kindly provided by Dr. J. Cammenga). cDNA shRNA MISSION™ interference lentiviral vectors targeting *Hif-1α* or non-targeting shRNA scramble control were designed by the RNAi Consortium (TRC) at the Broad Institute and purchased from Sigma. The shRNAs were subcloned to the lentiviral vector pLKO.1-EGFP (kindly provided by Dr. J. Larsson, Lund). Virus supernatants were obtained by calcium phosphate transfection of 293T cells together with the helper plasmids pMD.G and pCMVΔR8.2 (lentivirus) or pVSV-G and pGAG-pol (retrovirus), as previously described (9). Cells were transduced by using Retronectin™ (T100/A; Takara, Tokyo) and spinfection at 1800 g in the presence of 5 μg Polybrene/mL (H9268; Sigma-Aldrich). GFP^+^ cells were sorted on an FACSAria (BD Biosciences). The following shRNA sequences were used in the pLKO.1-GFP lentiviral vector:

Hif-1α (TRC number TRCN0000054450): CCGGCCAGTTACGATTGTGAAGTTACTCGAGTAACTTCACAATCGTAACTGGTTTTTG
Scramble (TRC number SHC002): CCGGCCTAAGGTTAAGTCGCCCTCGCGAGGGCGACTTAACCTTAGGTTTTG

### Western blot analysis

FDCP1 cells (3 × 10^6^) were washed in PBS and lysed in buffer containing 25 mM Tris pH 7.5, 150 mM NaCl, 1 mM dithiothreitol (DTT), 1 mM ethylenediaminetetraacetic acid, 1% Triton X-100, and cOmplete Protease Inhibitor Cocktail (11697498001; Roche, Basel, Switzerland). Lysates of 0.5 × 10^6^ cells were separated on a 4–12% NuPAGE bis-tris gel (NP0322BOX; Thermo Fisher Scientific) and blotted to PVDF membranes (RPN303F; GE Healthcare, Buckinghamshire, UK). Immunoblotting was performed overnight at 4°C with primary rabbit anti-HIF-2α (NB100-122; Novus Biologicals, Littleton, CO) antibody and horseradish peroxidase (HRP)-coupled secondary donkey anti-rabbit antibody (711-036-152; Jackson Immunoresearch, Cambridgeshire, UK) for 1 h. Glyceraldehyde-3-phosphate dehydrogenase (MAB374, GAPDH; Chemicon, Temecula, CA) antibody was used as a control together with the HRP-coupled secondary goat anti-mouse antibody (115-035-062; Jackson Immunoresearch), and the molecular weight marker was PageRuler™ Plus from Fermentas (26619; Thermo Fisher Scientific). c-kit^+^ (2.5 × 10^6^) was washed with PBS and lysed with NE-PER nuclear and cytoplasmic extraction reagents (78833; Thermo Fisher Scientific) according to the manufacturer's protocol. Cytoplasmic lysates of 0.4 × 10^6^ cells were separated on a 4–12% NuPAGE bis-tris gel (NP0322BOX; Thermo Fisher Scientific) and blotted to PVDF membranes (RPN303F; GE Healthcare). Immunoblotting was performed overnight at 4°C with primary rabbit anti-p65 pSer536 (NB100-82088; Novus Biologicals) antibody and HRP-coupled secondary donkey anti-rabbit antibody (711-036-152; Jackson Immunoresearch) for 1 h. Glyceraldehyde-3-phosphate dehydrogenase (MAB374, GAPDH; Chemicon) antibody was used as a control together with the HRP-coupled secondary goat anti-mouse antibody (115-035-062; Jackson Immunoresearch), and the molecular weight marker was Precision plus WesternC standards (161-0376; Biorad, Hercules, CA) with Precision Protein™ StrepTactin-HRP conjugate (161-0381; Biorad). Blots were visualized with Pierce ECL Plus (32132; Thermo Fisher Scientific).

### *In vivo* reconstitution assay

LSK cells isolated from CD45.1 mice were cultured for 2 days in normoxia or hypoxia with BSO. For transplantation, the culture equivalent to 2000 initially plated LSK cells along with 2 × 10^5^ freshly isolated BM supporter cells from CD45.2 mice were injected in the lateral tail vein of lethally irradiated (9 Gy) CD45.2 mice. Peripheral blood was collected by lateral tail vein bleeding post-transplant, and red blood cells were lysed by using ammonium chloride. Blood cells were stained with CD45.1-PE (110707, A20) and CD45.2-FITC (109805, 104), and with PECy5-conjugated Gr1/Mac-1 or CD19/B220 (all from BioLegend) for detection of myeloid or lymphoid cells, respectively.

### Quantitative RT-PCR

Total RNA was isolated from LSK cells by using RNeasy^®^ Micro Kit (74004; Qiagen, Hilden, Germany). cDNA was generated by annealing total RNA to random primers (60 min at 50°C and 15 min at 70°C) in a reaction mixture containing 240 ng of random primer (P/N 58875), 0.5 mM dNTP (R0192; Thermo Fisher Scientific), 2 U/μL RNaseOUT (P/N 100000840), and 5 U/μL SuperscriptIII reverse transcriptase, 1 × first-strand buffer, and 5 μM DTT (P/N 56575) (all reagents from Invitrogen, Thermo Fisher Scientific unless stated otherwise). To quantify transcripts, reactions were performed in 10 μL with 2 × SYBR green master mix (04913850001; Roche), 0.5 μM of forward and reverse primers, or FastStart Universal Probe Master (Rox) (Roche), 20 × Assays-on-Demand™ probes (Applied Biosystems, Foster City, CA), RNase-free water, and 4–12 ng template. β-actin or Hprt1 was used for sample normalization. All samples were set in triplicates, and non-template controls were used for all samples. qRT-PCR was initiated by holding for 10 min at 95°C and 40 cycles of 15 s at 95°C and 60 s at 60°C, and reactions were performed by using 7900 HT Fast Real-time PCR System (Applied Biosystems). Relative expression was calculated by normalization to the reference gene. The following primers were used for qRT-PCR:

*Hif-1α*-forward: 5′-TGACGGCGACAGGGTTTACA-3′
*Hif-1α*-reverse: 5′-AATATGGCCCGTGCAGTGAA-3′
*Hif-2α*-forward: 5′-TGACCCAAGACGGTGACATGA-3′
*Hif-2α*-reverse: 5′-CATGGTCGCAAGGATGAGTGA-3′
*Cat*-forward: 5′-TCAGAAGAAAGCGGTCAAGAATT-3′
*Cat*-reverse: 5′-GGATGCGGGCCCCATA-3′
*Sod*-forward: 5′-GCTGCACCACAGCAAGCA-3′
*Sod*-reverse: 5′-TCGGTGGCGTTGAGATTGT-3′
β-actin-forward: 5′-GCTGTATTCCCCTCCATCGTG-3′
β-actin-reverse: 5′-CACGGTTGGCCTTAGGGTTCAG-3′
The following probes (assay ID) were used for qRT-PCR:
*Pdk1*: Mm00554306_m1
*Vegfa*: Mm00437306_m1
*Hk1*: Mm00439344_m1
*Prdx4*: Mm00450261_m1
*Hprt1*: Mm00446968_m1

### RNA extraction and microarray analysis

Total RNA was extracted from LSK cells, either freshly sorted or cultured in normoxia or hypoxia for 24 or 48 h, and stabilized in RNAprotect buffer according to the “Purification of total RNA from animal and human cells” protocol of the RNeasy Plus Micro Kit from Qiagen (74034). Sample processing was performed at an Affymetrix Service Provider and Core Facility, “KFB—Center of Excellence for Fluorescent Bioanalytics” (Regensburg, Germany). In brief, cells were stored and shipped in RNAprotect buffer at 2–8°C. After pelleting, the RNAprotect buffer was replaced by RLT Plus and the samples were homogenized by vortexing for 30 s. Genomic DNA contamination was removed by using gDNA Eliminator spin columns. Next, ethanol was added and the samples were applied to RNeasy MinElute spin columns followed by several wash steps. Finally, total RNA was eluted in 12 μL of nuclease-free water. Purity and integrity of the RNA was assessed on the Agilent 2100 Bioanalyzer with the RNA 6000 Pico LabChip reagent set (Agilent, Palo Alto, CA). Sample preparation for microarray hybridization was carried out as described in the NuGEN Ovation PicoSL WTA System V2 and NUGEN Encore Biotin Module manuals (NuGEN Technologies, Inc., San Carlos, CA). In brief, 15 ng of total RNA was reverse transcribed into double-stranded cDNA in a two-step process, introducing a SPIA tag sequence. Bead-purified cDNA was amplified by an SPIA amplification reaction followed by an additional bead purification. Then, 4.5 μg of SPIA cDNA was fragmented, terminally biotin-labeled, and hybridized to Affymetrix Mouse Gene 2.0 ST arrays for 16 h at 45°C in a GeneChip hybridization oven 640. Hybridized arrays were washed and stained in an Affymetrix Fluidics Station FS450, and the fluorescent signals were measured with an Affymetrix GeneChip Scanner 3000 7G. Fluidics and scan functions were controlled by the Affymetrix GeneChip Command Console v4.1.3 software. Summarized probe set signals in log2 scale were calculated by using the Robust Multiarray Average (18) algorithm, and differentially expressed probes were identified by using limma (48). These probes were then clustered into 20 partitions after hierarchical clustering by cutting the dendrogram using the *cutree* function to produce a supervised clustering of the data.

### Pathway analysis by IPA

The core analysis function included in the IPA software (Qiagen) (including biological processes, canonical pathways, upstream transcriptional regulator, and gene networks) was used to interpret the common differentially expressed genes (both up- and downregulated genes) between LSK cells cultured in normoxia or hypoxia for 24 or 48 h, respectively. Datasets containing gene identification and corresponding expression values were uploaded into the IPA software. Each gene was mapped to its corresponding gene object in the Ingenuity Pathway Knowledge Base.

### Evaluation of NF-κB activity by lentiviral GFP reporter construct

The Cignal reporter and NF-κB Cignal lenti reporter (GFP) assay kits were purchased from Qiagen (CLS-PCG and CLS-013G). Transient infection of 1 × 10^4^ cells/well of LSK cells from mouse BM with Cignal lentiviral particles at the multiplicity of infection 50 and Polybrene (5 μg/mL; Sigma-Aldrich) were generated by spinfection at 1800 g. Forty-eight hours after infection, the cells were split into different cultures and incubated for an additional 48 h in normoxia or hypoxia. As a positive control, 100 ng/mL murine tumor necrosis factor-α (315-01A; Peprotech) was used. Cells were analyzed by flow cytometry for the number of GFP-expressing cells on FACSCanto (BD Biosciences).

### Evaluation of NF-κB activity in nuclear extracts

NF-κB activity was measured in nuclear protein extracts from 5 × 10^5^ c-kit^+^ BM cells isolated by magnetic selection and incubated for 24 h in normoxia or hypoxia. The TransAM™ NF-κB p65 protein assay (40096; Active Motif, Carlsbad, CA), an ELISA-based method designed to specifically detect and quantify NF-κB p65 subunit activation, with high sensitivity and reproducibility, was used according to the manufacturer's protocol and analyzed at 450 nm with a reference at 650 nm by using a microplate absorbance reader Biorad 680 (Biorad).

### Statistical analysis

Data are expressed as the mean or mean ± standard deviation. Statistical analysis was performed by using Student's *t*-test, Tukey's test (comparing all samples with each other), Dunnett's test (comparing samples with a control), Dunn's test (non-parametric test comparing samples with a control), Mann–Whitney *U*-test, and Wilcoxon signed-rank test by GraphPad Prism 7 (GraphPad Software, Inc., La Jolla CA).

## Supplementary Material

Supplemental data

Supplemental data

Supplemental data

Supplemental data

Supplemental data

Supplemental data

Supplemental data

Supplemental data

Supplemental data

Supplemental data

Supplemental data

## Abbreviations Used

7AAD: 7-Aminoactinomycin D
AxV: Annexin V
BM: bone marrow
BSO: DL-buthionine-(S,R)-sulfoximine
CAT: catalase
cDNAs: complementary DNAs
CMP: common myeloid progenitor
CO_2_: carbon dioxide
DCF: 2′,7′-dichlorofluorescein
DMOG: dimethyloxalylglycine
ELISA: enzyme-linked immunosorbent assay
GFP: green fluorescent protein
GSH: glutathione
H: hypoxia
H_2_DCFDA: 6-carboxy-2′,7′-dichlorodihydrofluorescein diacetate
H_2_O_2_: hydrogen peroxide
HIF: hypoxia-inducible factor
HMOX: heme oxygenase 1
HRP: horseradish peroxidase
HSC: hematopoietic stem cell
HSPCs: hematopoietic stem cell and progenitor cells
IL: interleukin
IPA: Ingenuity Pathway Analysis
LSK: Lineage-Sca-1^+^c-kit^+^
LT: long-term
MFI: mean fluorescence intensity
MPP: multipotent progenitor
N: normoxia
NAC: N-acetyl-L-cysteine
NF-κB: nuclear factor-kappa B
NRF2: nuclear factor erythroid 2-related factor 2
O_2_: oxygen molecule
^•^O_2_^−^: superoxide anion radical
^•^OH: hydroxyl radical
PBS: phosphate-buffered saline
PHD: prolyl hydroxylase domain
PI: propidium iodide
pIpC: polyI:polyC
PQ: paraquat
qRT-PCR: quantitative real-time PCR
ROS: reactive oxygen species
SCF: stem cell factor
SD: standard deviation
shRNA: short hairpin RNA
SOD: superoxide dismutase
ST: short-term
TPO: thrombopoietin
