# Supplementary material for: Putative Role of Nuclear Factor-Kappa B But Not Hypoxia-Inducible Factor-1α in Hypoxia-Dependent Regulation of Oxidative Stress in Hematopoietic Stem and Progenitor Cells
Source: Antioxid Redox Signal. 2019 Jun 20;31(3):211–26. doi: 10.1089/ars.2018.7551 (PMC6590716; doi:10.1089/ars.2018.7551)
Supplement: Supplemental data [file Supp_Fig1.pdf]

Supplementary Data

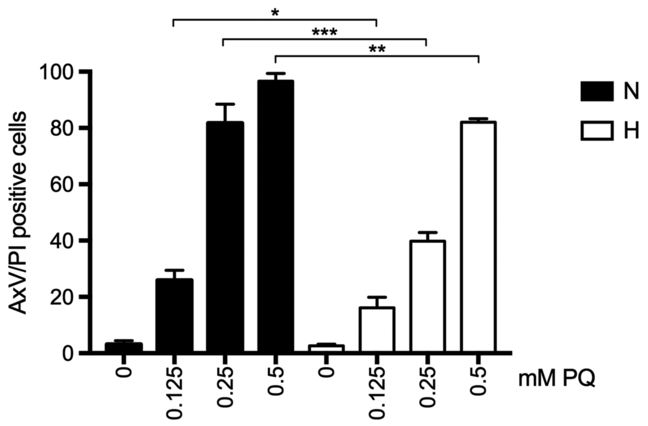

SUPPLEMENTARY FIG. S1. Hypoxic treatment protects LSK BM cells against PQ-mediated cytotoxicity. Flow cytometric evaluation of cell viability by AxV and PI staining of LSK cells cultured in N or H for 48 h with or without PQ (0.125, 0.25, and 0.5 mM). Data are presented as mean ± SD ($n = 5$). Statistical analysis was performed by using paired Student's *t*-test. $*p < 0.05$, $**p < 0.01$, and $***p < 0.001$. AxV, Annexin V; BM, bone marrow; H, hypoxia; LSK, Lineage-Sca-1$^+$c-kit$^+$; N, normoxia; PI, propidium iodide; PQ, paraquat; SD, standard deviation.
