# Supplementary material for: Putative Role of Nuclear Factor-Kappa B But Not Hypoxia-Inducible Factor-1α in Hypoxia-Dependent Regulation of Oxidative Stress in Hematopoietic Stem and Progenitor Cells
Source: Antioxid Redox Signal. 2019 Jun 20;31(3):211–26. doi: 10.1089/ars.2018.7551 (PMC6590716; doi:10.1089/ars.2018.7551)
Supplement: Supplemental data [file Supp_Fig2.pdf]

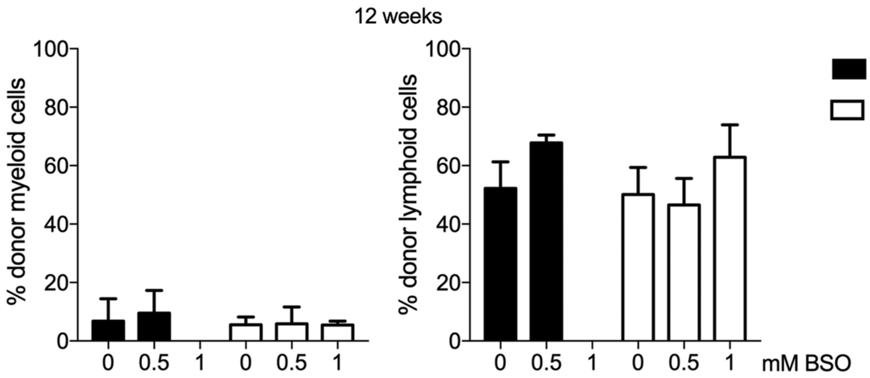

SUPPLEMENTARY FIG. S2. Myeloid and lymphoid repopulation ability of HSPCs under oxidative stress. Non-competitive repopulation assay with LSK cells cultured with increasing BSO concentrations for 48 h in H or N. Cultured cells equivalent to 2000 initially plated LSK cells (CD45.1) along with 2×10^5 BM supporter cells (CD45.2) were transplanted into lethally irradiated (9 Gy) CD45.2 recipient mice. Lineage distribution of donor-derived (CD45.1) myeloid cells (Gr1/Mac-1) and lymphoid cells (CD19/B220) in the peripheral blood of recipient mice (CD45.2) was analyzed by flow cytometry at 12 weeks after BM transplantation. Data are presented as mean ± SD ($n = 3$–4/group). BSO, DL-buthionine-(S,R)-sulfoximine; HSPCs, hematopoietic stem cell and progenitor cells.
