# Supplementary material for: Putative Role of Nuclear Factor-Kappa B But Not Hypoxia-Inducible Factor-1α in Hypoxia-Dependent Regulation of Oxidative Stress in Hematopoietic Stem and Progenitor Cells
Source: Antioxid Redox Signal. 2019 Jun 20;31(3):211–26. doi: 10.1089/ars.2018.7551 (PMC6590716; doi:10.1089/ars.2018.7551)
Supplement: Supplemental data [file Supp_Fig3.pdf]

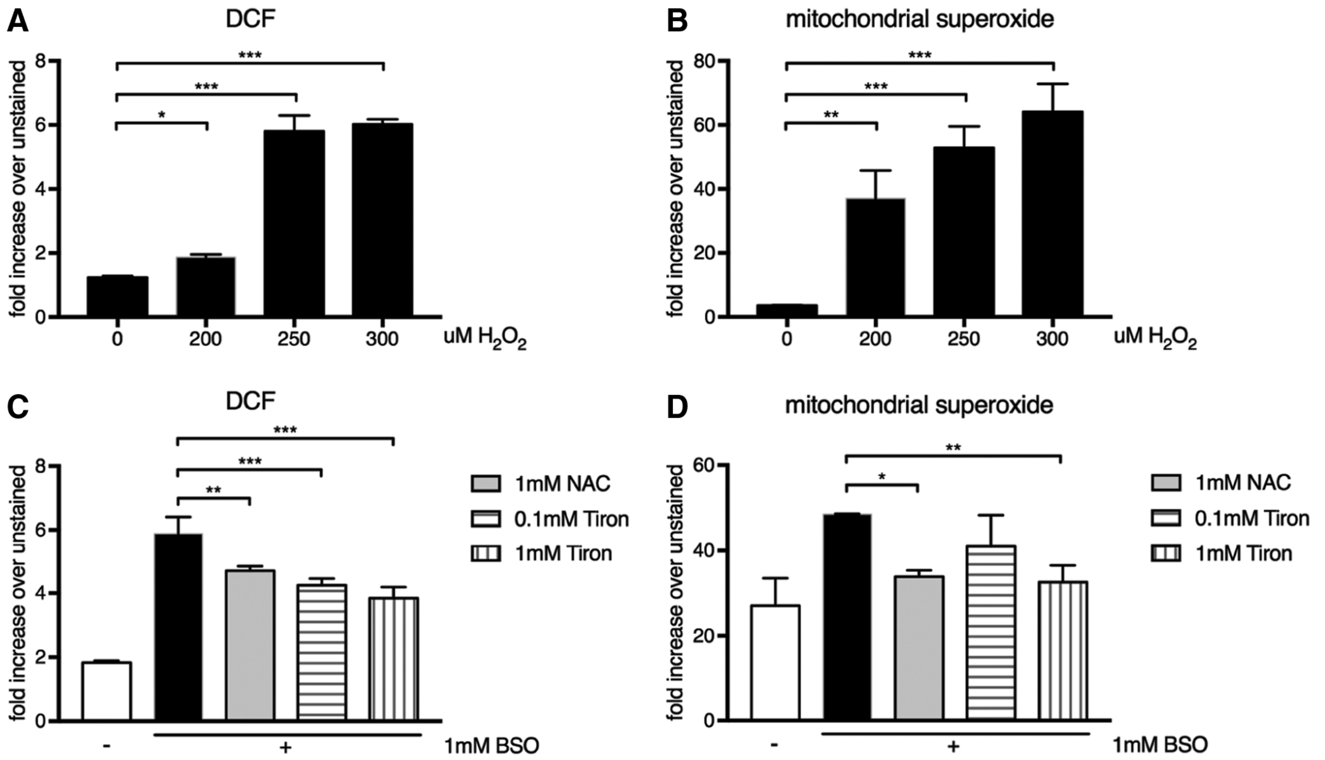

SUPPLEMENTARY FIG. S3. Flow cytometric validation of ROS after incubation with H_2O_2 or antioxidants NAC and Tiron. **(A, B)** LSK cells were cultured in N for 1 h with or without H_2O_2 (200, 250, and 300 μM). Intracellular level of ROS in H_2DCFDA-stained **(A)** or of mitochondrial superoxide in MitoSOX Red-stained **(B)** cells. **(C, D)** LSK cells were cultured in N for 24 h with or without BSO (1 mM) and antioxidants NAC (1 mM) or Tiron (0.1 and 1 mM). Intracellular level of ROS in H_2DCFDA-stained **(C)** or of mitochondrial superoxide in MitoSOX Red-stained **(D)** cells. Measurements were normalized to PBS-treated cells (no H_2DCFDA or MitoSOX). Data are presented as mean ± SD ($n = 3$). Statistical analysis was performed by using Dunnett's test to sample without H_2O_2 **(A, B)** or with 1 mM BSO and without antioxidant **(C, D)**. *$p < 0.05$, **$p < 0.01$, and ***$p < 0.001$. DCF, 2′,7′-dichlorofluorescein; H_2DCFDA, 6-carboxy-2′,7′-dichlorodihydrofluorescein diacetate; H_2O_2, hydrogen peroxide; NAC, N-acetyl-L-cysteine; PBS, phosphate-buffered saline; ROS, reactive oxygen species.
