# Supplementary material for: Putative Role of Nuclear Factor-Kappa B But Not Hypoxia-Inducible Factor-1α in Hypoxia-Dependent Regulation of Oxidative Stress in Hematopoietic Stem and Progenitor Cells
Source: Antioxid Redox Signal. 2019 Jun 20;31(3):211–26. doi: 10.1089/ars.2018.7551 (PMC6590716; doi:10.1089/ars.2018.7551)
Supplement: Supplemental data [file Supp_Fig4.pdf]

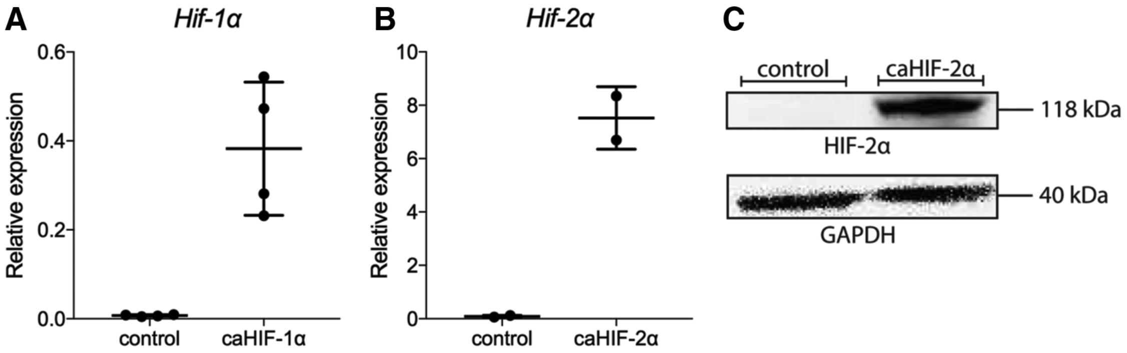

SUPPLEMENTARY FIG. S4. Constitutively active HIF-1α and HIF-2α protein is expressed in N. **(A)** qRT-PCR analysis of *Hif-1*α expression in LSK cells transduced with caHIF-1α or empty vector (control), 24 h after culture in N. LSK cells were sorted for GFP expression 2 days after transduction. Data were normalized to β-*actin* expression. Each *dot* represents one sample, and data are presented as mean ± SD ($n = 4$, in triplicates). **(B)** qRT-PCR analysis of *Hif-2*α *expression* in LSK cells transduced with caHIF-2α or empty vector (control), 24 h after culture in N. LSK cells were sorted for GFP expression 2 days after transduction. Data were normalized to β-*actin* $\times 10^{-3}$ expression. Each *dot* represents one sample, and data are presented as mean ± SD ($n = 2$, in triplicates). **(C)** Protein levels of HIF-2α in FDCP1 cells transduced with GFP-containing lentiviral caHIF-2α were analyzed with Western blot. Loading control used was antibody against the house-keeping protein GAPDH. Full blot in Supplementary Figure S10. GFP, green fluorescent protein; HIF, hypoxia-inducible factor; qRT-PCR, quantitative real-time PCR.
