# Supplementary material for: Putative Role of Nuclear Factor-Kappa B But Not Hypoxia-Inducible Factor-1α in Hypoxia-Dependent Regulation of Oxidative Stress in Hematopoietic Stem and Progenitor Cells
Source: Antioxid Redox Signal. 2019 Jun 20;31(3):211–26. doi: 10.1089/ars.2018.7551 (PMC6590716; doi:10.1089/ars.2018.7551)
Supplement: Supplemental data [file Supp_Fig10.pdf]

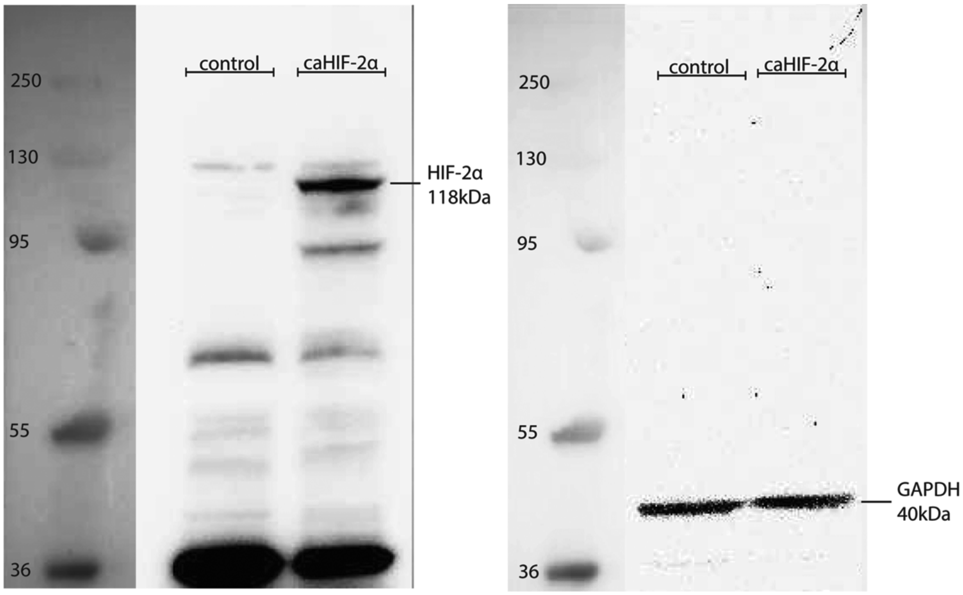

SUPPLEMENTARY FIG. S10. Full scanned Western blot shown in Supplementary Figure S4C. Protein levels of HIF-2α in FDCP1 cells transduced with GFP-containing lentiviral caHIF-2α were analyzed with Western blot. Loading control used was antibody against the house-keeping protein GAPDH. The protein bands (chemiluminescent protein detection) have been merged with the molecular weight markers (chromogenic detection).
