# Supplementary material for: Putative Role of Nuclear Factor-Kappa B But Not Hypoxia-Inducible Factor-1α in Hypoxia-Dependent Regulation of Oxidative Stress in Hematopoietic Stem and Progenitor Cells
Source: Antioxid Redox Signal. 2019 Jun 20;31(3):211–26. doi: 10.1089/ars.2018.7551 (PMC6590716; doi:10.1089/ars.2018.7551)
Supplement: Supplemental data [file Supp_Fig5.pdf]

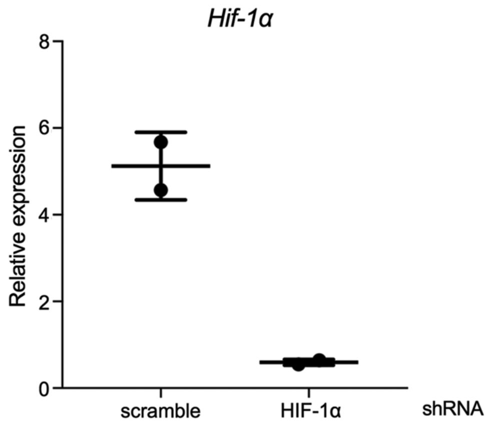

SUPPLEMENTARY FIG. S5. Efficient knockdown of *Hif-1*α RNA by lentiviral shRNA to HIF-1α in LSK cells. qRT-PCR analysis of *Hif-1*α expression in LSK cells transduced with lentiviral shRNA to *Hif-1*α or scramble control in LSK cells sorted for GFP expression 2 days after transduction. Data were normalized to β-*actin* $\times 10^{-3}$ expression. Each *dot* represents one sample, and data are presented as mean ± SD ($n = 2$, in triplicates). shRNA, short hairpin RNA.
