# Supplementary material for: Putative Role of Nuclear Factor-Kappa B But Not Hypoxia-Inducible Factor-1α in Hypoxia-Dependent Regulation of Oxidative Stress in Hematopoietic Stem and Progenitor Cells
Source: Antioxid Redox Signal. 2019 Jun 20;31(3):211–26. doi: 10.1089/ars.2018.7551 (PMC6590716; doi:10.1089/ars.2018.7551)
Supplement: Supplemental data [file Supp_Fig6.pdf]

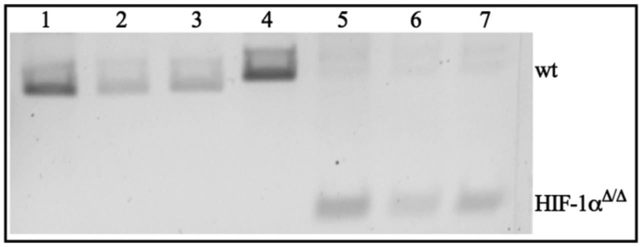

SUPPLEMENTARY FIG. S6. ***pIpC injections of Mx1-Cre:HIF-1α$^{flox/flox}$*** **mice induce expression of Cre recombinase and removal of *Hif-1α* gene.** PCR analysis on agarose-gel stained with Gel Red to confirm the conditional knockout of *Hif-1*α in mice treated with pIpC. The wt band is 1.2 kbp, and the deleted band is 300 bp. Lanes 1–4, *Mx1-Cre* mice; lanes 5–7, *HIF-1*α$^{\Delta/\Delta}$ mice. pIpC, polyI:polyC; wt, wild-type.
