# Supplementary material for: Putative Role of Nuclear Factor-Kappa B But Not Hypoxia-Inducible Factor-1α in Hypoxia-Dependent Regulation of Oxidative Stress in Hematopoietic Stem and Progenitor Cells
Source: Antioxid Redox Signal. 2019 Jun 20;31(3):211–26. doi: 10.1089/ars.2018.7551 (PMC6590716; doi:10.1089/ars.2018.7551)
Supplement: Supplemental data [file Supp_Fig7.pdf]

A

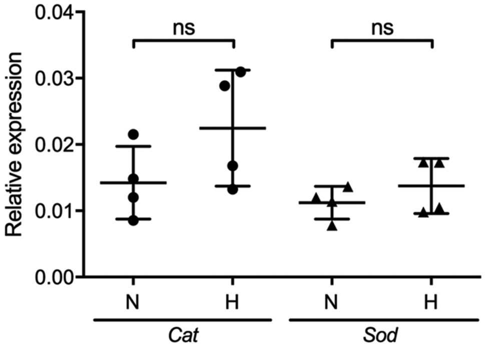

B

Probe	ID	logFC	t	P.Value	adj.P.Val	B
**24h H vs N**						
17388803	Cat	0.522848527455908	2.73218395441908	0.017342971955842	0.488899737858147	-3.2283100076163
17548153	Cat	0.456191220872072	2.0319241475287	0.0635060044143466	0.676455046453781	-4.40448601626768
17333347	Sod2	0.0706901346876894	0.690482373262509	0.502249273415138	0.94116834307447	-6.00982718393724
**48h H vs N**						
17548153	Cat	0.778763045667665	3.46869331389187	0.00425926079998863	0.113266892557066	-2.07883568853695
17388803	Cat	0.640011084093763	3.34442562766671	0.00539664038620488	0.129647935367601	-2.3082387232747
17333347	Sod2	-0.293870776836121	-2.87045133410446	0.0133346930957083	0.20381518786256	-3.17765300784439

SUPPLEMENTARY FIG. S7. Antioxidant enzymes *Cat* and *Sod* are not upregulated by H. (A) qRT-PCR analysis of *Cat* and *Sod* expression in LSK cells, 24 h after culture in N or H. Data were normalized to β-*actin* expression. Each *dot* represents one sample, and data are presented as mean ± SD ($n = 4$, in triplicates). Statistical analysis was performed by using paired Student's *t*-test. **(B)** Microarray analysis of *Cat* and *Sod*, 24 or 48 h after culture in N or H. The adjusted *p*-value is based on the comparison of H *versus* N for 24 or 48 h, respectively, and it is significant if below the threshold of 0.05. *Cat*, catalase; ns, nonsignificant; *Sod*, superoxide dismutase.
