# Supplementary material for: Putative Role of Nuclear Factor-Kappa B But Not Hypoxia-Inducible Factor-1α in Hypoxia-Dependent Regulation of Oxidative Stress in Hematopoietic Stem and Progenitor Cells
Source: Antioxid Redox Signal. 2019 Jun 20;31(3):211–26. doi: 10.1089/ars.2018.7551 (PMC6590716; doi:10.1089/ars.2018.7551)
Supplement: Supplemental data [file Supp_Fig8.pdf]

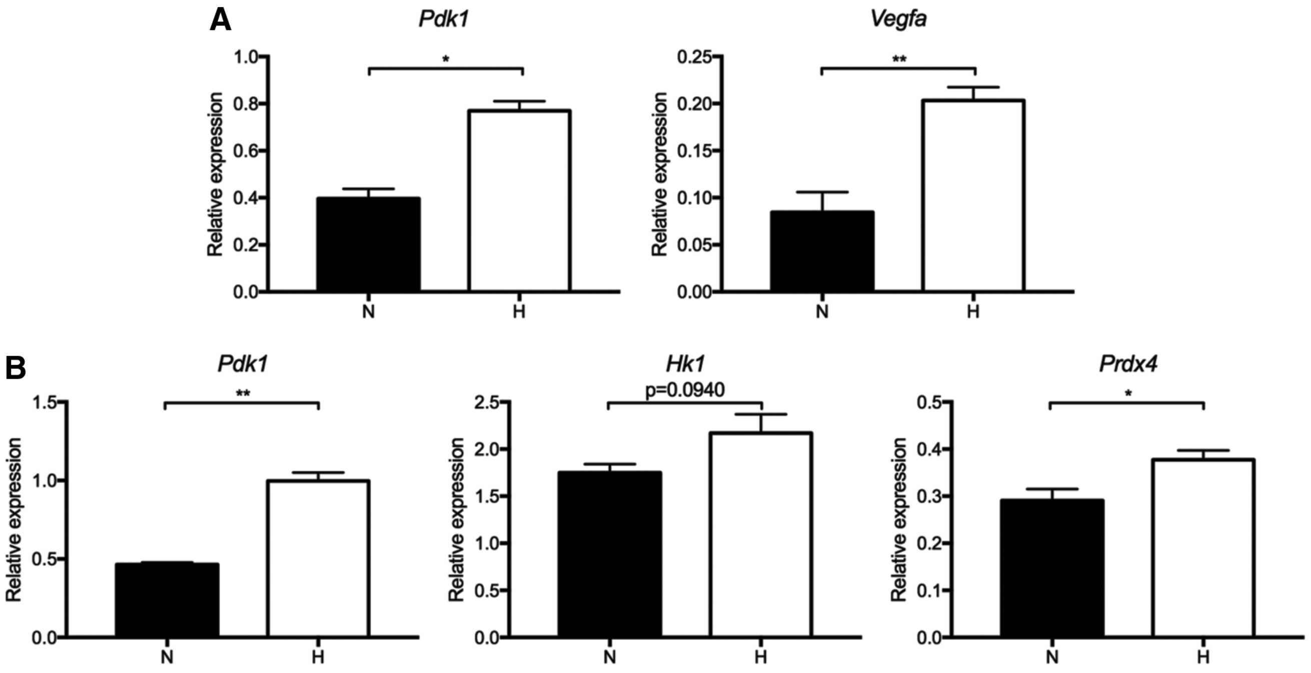

SUPPLEMENTARY FIG. S8. qRT-PCR confirms microarray data of upregulated or maintained genes in H. (A, B) qRT-PCR analysis of *Pdk1* and *Vegfa* expression in LSK cells 24 h **(A)** and *Pdk1*, *Hk1*, and *Prdx4* expression in LSK cells 48 h **(B)** after culture in N or H. Data were normalized to *Hprt1* expression. Each dot represents one sample, and data are presented as mean ± SD ($n = 3$, in triplicates). Statistical analysis was performed by using paired Student's *t*-test. *$p < 0.05$, **$p < 0.01$.
