# Supplementary material for: Putative Role of Nuclear Factor-Kappa B But Not Hypoxia-Inducible Factor-1α in Hypoxia-Dependent Regulation of Oxidative Stress in Hematopoietic Stem and Progenitor Cells
Source: Antioxid Redox Signal. 2019 Jun 20;31(3):211–26. doi: 10.1089/ars.2018.7551 (PMC6590716; doi:10.1089/ars.2018.7551)
Supplement: Supplemental data [file Supp_Fig11.pdf]

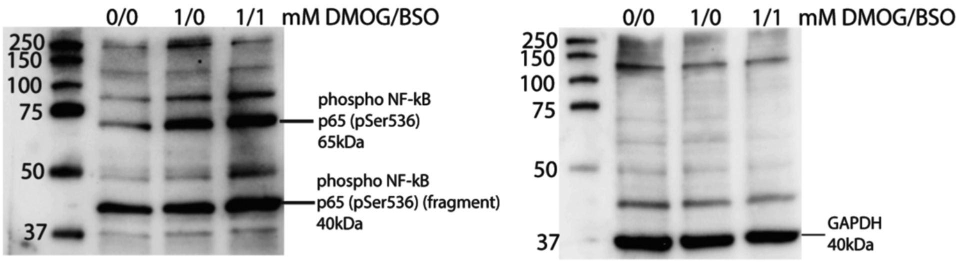

SUPPLEMENTARY FIG. S11. Full scanned Western blots shown in Figure 6B. Cytoplasmic protein levels of NF-κB p65 in c-kit$^+$ cells treated with or without 1 mM DMOG and 1 mM BSO for 4 h in N were analyzed with Western blot. Loading control used was antibody against the house-keeping protein GAPDH. The antibody against p65 (pSer536) detects a band at 65 kDa, whereas the band at $\sim$40 kDa is of unknown origin but could be a degradation product of NF-κB. DMOG, dimethyloxalylglycine; NF-κB, nuclear factor kappa B.
