# Supplementary material for: Putative Role of Nuclear Factor-Kappa B But Not Hypoxia-Inducible Factor-1α in Hypoxia-Dependent Regulation of Oxidative Stress in Hematopoietic Stem and Progenitor Cells
Source: Antioxid Redox Signal. 2019 Jun 20;31(3):211–26. doi: 10.1089/ars.2018.7551 (PMC6590716; doi:10.1089/ars.2018.7551)
Supplement: Supplemental data [file Supp_Fig9.pdf]

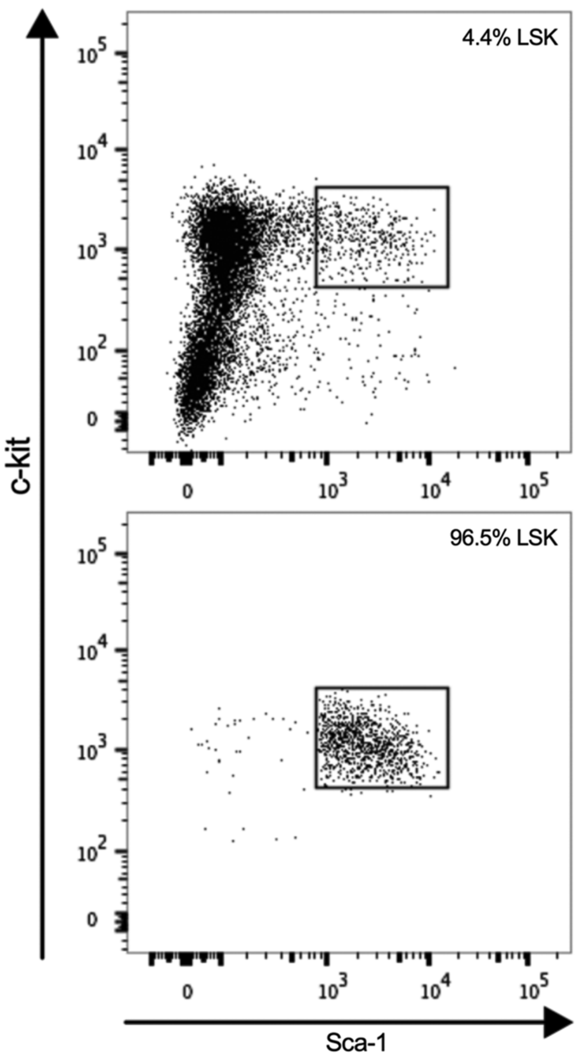

SUPPLEMENTARY FIG. S9. Representative reanalysis of sorted LSK cells. Before sorting, the initial amount of LSK cells was 4.4%, and after sorting it was 96.5%. As another example the 10 latest sortings showed the following purities in percent: 97, 96.5, 96.2, 96.6, 96.5, 98, 97, 98, 96, and 98.
